# Deep learning for the change-point Cox model with current status data

**DOI:** 10.1007/s10985-026-09689-y

**Published:** 2026-02-09

**Authors:** Qiyue Huang, Anyin Feng, Qiang Wu, Xingwei Tong

**Affiliations:** 1 School of Statistics, Beijing Normal University, Beijing, China; 2 Department of Applied Mathematics, The Hong Kong Polytechnic University, Hong Kong, China; 3 Department of Statistics, Faculty of Arts and Science, Beijing Normal University at Zhuhai, Zhuhai, China

**Keywords:** Change point, Current status data, Deep neural network, Semiparametric efficiency

## Abstract

This study develops estimation methods for a deep partially linear Cox proportional hazards model with a change point under current status data, aiming to accommodate complex change-point effects. Prior work has largely relied on linear models, which may inadequately capture relationships among multivariate covariates and thus hinder accurate change-point detection. To address this, we use a deep neural network to model covariate effects within the Cox framework and propose a maximum likelihood estimation procedure for the model. We establish asymptotic properties of the resulting estimators, including consistency, asymptotic independence, and semiparametric efficiency. Simulation studies indicate that the proposed inference procedure performs well in finite samples. An analysis of a breast cancer dataset is provided to illustrate the methodology.

**Supplementary Information:**

The online version contains supplementary material available at 10.1007/s10985-026-09689-y.

## Introduction

A change-point model is a statistical framework for detecting points or periods at which the properties of the data shift abruptly (Pons 2003; Deng et al. 2017, 2022). Such models are vital across many domains, including economics (Li et al. 2024), environmental science, signal processing, and medical settings (Deng et al. 2022), where identifying when changes occur is crucial for decision-making and subsequent analysis.

Many studies have examined change-point models in survival analysis (Loader 1991; Pons 2003; Dupuy 2006; Kosorok and Song 2007; Torben and Scheike 2007; Oueslati and Lopez 2013; Deng et al. 2022). Among these, the Cox proportional hazards model has attracted the most attention. Luo and Boyett (1997) first considered the model $\Lambda (t)=\Lambda _0(t)\exp (\beta _0I_{\{X\le \zeta _0\}}+\alpha _0 Z)$, where the covariates *X* and *Z* are one-dimensional and a constant term $\beta _0$ is added when the threshold $\zeta _0$ is reached. Pons (2003) considered the Cox model $\Lambda (t)=\Lambda _0(t)\exp \{{\boldsymbol{\alpha }}_0^\top {\boldsymbol{Z}}_1+{\boldsymbol{\beta }}_0^\top {\boldsymbol{Z}}_2I_{\{Z_3\le \zeta _0\}}+{\boldsymbol{\gamma }}_0^\top {\boldsymbol{Z}}_2I_{\{Z_3>\zeta _0\}}\}$, where the covariate jumps at a threshold $\zeta _0$, and showed that the change-point estimator is *n*-consistent. Kosorok and Song (2007) and Song et al. (2009) extended the asymptotic properties of the Cox model to a class of transformation models and introduced a maximal score-based test for the existence of a change point using random shuffling. Lee et al. (2020) further studied tests for the change-point effect with right-censored data and proposed three maximum Wald or score-based procedures that are important for model identification. More recently, for right-censored data, Deng et al. (2022) extended the Cox model with a change point to one with a change hyperplane. They developed a computational method for this model, proved the convergence of the change hyperplane, and derived the asymptotic distribution of the estimators.

Most existing methods focus on right-censored data. In this type of data, some individuals have fully observed failure times, while others are right-censored, known only after the time of censoring. However, in some clinical settings, individuals scheduled for observation to detect clinically significant changes in their health or disease status may miss some scheduled appointments and return with altered status (Ma et al. 2014), such as in studies of traumatic brain injury (Vanier et al. 2020). In such cases, change-point models developed for right-censored data may fail because the event time of interest can be left-censored or right-censored. To overcome this limitation, we study a change-point model for “case-I” interval-censored data, also known as current status data.

Current status data arise when the exact event time is unobserved and only the occurrence status at a single examination is recorded (Huang 1996; Huang and Wellner 1997; Sun 2006). Compared with right-censored data, the precise value of the survival time *T* is unknown, making statistical inference with current status data more challenging. Huang (1996) first established the Cox model framework for current status data and derived its asymptotic properties. For regression analysis, a partially linear proportional hazards model was later proposed (Lu and McMahan 2018).

However, in clinical studies, some covariates can exert complex nonlinear interaction effects on the logarithm of the cumulative hazard of the failure time *T*. When interaction effects are present, simple linear or purely additive models may fail to capture them, leading to substantial bias. To address this, Wu et al. (2024) proposed a deep learning approach that flexibly models the nonparametric effects of multivariate covariates and their potential relationships. The partially linear Cox model is then formulated as

1 $$\begin{aligned} \Lambda (t|{\boldsymbol{X}}, {\boldsymbol{Z}}) = \Lambda _0(t)\exp \{{\boldsymbol{\beta }}_0^\top {\boldsymbol{X}}+g_0({\boldsymbol{Z}})\} , \end{aligned}$$

where $\Lambda _0$ denotes the baseline cumulative hazard function, ${\boldsymbol{\beta }}_0\in \mathbb {R}^p$ is an unknown parameter vector, and $g_0: \mathbb {R}^r\rightarrow \mathbb {R}$ represents an unknown smooth function.

Although many methods have been developed for failure-time data with change points, most are designed for right-censored data and are not directly applicable to interval-censored settings. We therefore study a partially linear Cox model with a change point for current status data.

Our main contributions are summarized below:

(i) We introduce a deep partially linear Cox model with a change point for current status data and provide a practical estimation algorithm;
(ii) We establish consistency and convergence rates for the maximum likelihood estimators of all unknown components, including both parametric and nonparametric parts, derive the asymptotic normality of the parametric estimators, and attain semiparametric efficiency;
(iii) We develop a score-based testing procedure to assess the existence of a change point.

The remainder of the paper is organized as follows. Section 2 presents the estimation procedure for a change point in a partially linear Cox model with current status data. Section 3 provides theoretical results for the estimators, including consistency, convergence rates for all components, and the asymptotic normality of the parametric estimates. Section 4 introduces a score-based testing procedure to assess the presence of a change point. Section 5 reports numerical experiments, and Sect. 6 illustrates the method with the Rotterdam breast cancer data. Section 7 offers a summary and further discussion. All technical lemmas, necessary proofs, and computational details are provided in the supplementary material.

## Model specification and methodology

### Framework of deep neural network

Before constructing the model, we first outline the deep neural network framework. A DNN of depth $K\in {\mathbb {N}}^+$ and width ${\boldsymbol{p}}=(p_0,\dots ,p_{K+1})^\top$ is a composite function $g:{\mathbb {R}}^{p_0}\rightarrow {\mathbb {R}}^{p_{K+1}}$, expressed as

$$\begin{aligned} \begin{aligned} g(\textbf{z})&=W_Kg_K(\textbf{z})+\boldsymbol{\nu }_K,\\ g_K(\textbf{z})&=\boldsymbol{\sigma }(W_{K-1}g_{K-1}(\textbf{z})+\boldsymbol{\nu }_{K-1}),\dots ,g_1(\textbf{z}) =\boldsymbol{\sigma }(W_0\textbf{z}+\boldsymbol{\nu }_0), \end{aligned} \end{aligned}$$

where the matrices $W_k \in \mathbb {R}^{p_{k+1}\times p_k}$ and the vectors $\boldsymbol{\nu }_k \in \mathbb {R}^{p_{k+1}}$ ($k=0,\ldots ,K$) are the parameters to be estimated, and the activation function $\boldsymbol{\sigma }$ acts component-wise on vectors, i.e., for $\textbf{z}=(z_1,\ldots ,z_m)^\top$ we have $\boldsymbol{\sigma }(\textbf{z})=(\sigma (z_1),\ldots ,\sigma (z_m))^\top$. This study adopts the rectified linear unit (ReLU) activation function (Nair and Hinton 2010), defined as $\sigma (z)=\max \{0,z\}$. Compared with other activation functions, ReLU is computationally efficient, alleviates the vanishing gradient problem, and promotes stable gradient propagation in deep neural networks (Schmidt-Hieber 2020). For notational simplicity, let $\tilde{W}_k=(W_k,{\boldsymbol{\nu }}_k)\in {\mathbb {R}}^{p_{k+1}\times (p_k+1)}$. It follows from the definition of $g(\textbf{z})$ that *g* is also the function of the $\tilde{W}_k$ for $k=0,\dots ,K$, so we can rewrite $g(\textbf{z})$ as $g(\textbf{z};\tilde{W}_{k}'s)$.

The total number of parameters is $\sum _{k=0}^K p_{k+1}(p_k+1)$. Typically, an excessive number of parameters can induce overfitting. Weight pruning addresses this by reducing the total number of nonzero parameters, thereby yielding sparsely connected network layers (Schmidt-Hieber 2020). Given $D>0$ and $s\in {\mathbb {N}}^+$, we consider a sparsely connected neural network class

$$\begin{aligned} \begin{aligned} {\mathcal {G}}&:={\mathcal {G}}(K,{\boldsymbol{p}},s,D)\\&\quad = \left\{ g: g \text { is a DNN with }(K+1)\text { layers and width vector } \boldsymbol{p}\right. \\&\qquad \left. {\mid }\tilde{W}_k\in {\mathbb {R}}^{p_{k+1}\times (p_k+1)}, \Vert \tilde{W}_k\Vert _\infty \le 1, \text {for } k=0,\dots ,K, \sum _{k=0}^K\Vert \tilde{W}_k\Vert _0\le s,\Vert g\Vert _\infty \le D \right\} , \end{aligned} \end{aligned}$$

where $\Vert \cdot \Vert _\infty$ and $\Vert \cdot \Vert _0$ represent the $L^\infty$ norm and $L^0$ norm, respectively.

### Model and estimation

Consider a survival study involving *n* individuals with current status data. For each subject *i*, the event time of interest $T_i$ is not directly observed; instead, we observe a single examination time $U_i$ and a censoring indicator $\Delta _i=I_{\{T_i\le U_i \}}$, where $I_{\{\cdot \}}$ denotes the indicator function. Let ${\textbf{X}}_i$ be a *p*-dimensional covariate vector representing the treatments, which contains categorical explanatory variables and binary variables, ${\textbf{Z}}_i$ be a vector of *r*-dimensional covariate vector that affect the response in a nonparametric way, and $E_{i}\in {\mathbb {R}}$ denote the random variable of interest that exhibits a change point. Therefore, the observed data contains $\{(\Delta _i, U_i, {\textbf{X}}_i, {\textbf{Z}}_i, E_{i}), i=1,\dots ,n\}$.

Given a covariate vector $({\textbf{X}},{\textbf{Z}},E)\in {\mathbb {R}^{p+r+1}}$ , assume that the cumulative hazard function for the failure time *T* satisfies the following partial linear Cox model:

2 $$\begin{aligned} \Lambda (t|{\textbf{X}},{\textbf{Z}},E)=\Lambda _0(t)\exp \{{\boldsymbol{\beta }}_0^\top {\textbf{X}}+g_0({\textbf{Z}})+\{{\boldsymbol{\gamma }}_0^\top {\textbf{X}}+h_0({\textbf{Z}})\}I_{\{E> \zeta _0\}}\}, \end{aligned}$$

where $\Lambda _0(\cdot ):{\mathbb {R}}^+\cup \{0\}\rightarrow {\mathbb {R}}$ is the true baseline cumulative hazard function, ${\boldsymbol{\beta }}_0$, ${\boldsymbol{\gamma }}_0\in {\mathbb {R}}^p$ are true values of treatment effect vectors, $g_0(\cdot ): {\mathbb {R}}^r\rightarrow {\mathbb {R}}$ and $h_0(\cdot ):{\mathbb {R}}^r\rightarrow {\mathbb {R}}$ are true smooth functions, $\zeta _0\in {\mathbb {R}}$ is the true value of the change point. In this paper, we assume conditional independent censoring: $T \perp U \mid ({\textbf{X}},{\textbf{Z}},E)$. In addition, the distribution of $(U,{\textbf{X}},{\textbf{Z}},E)$ does not depend on $(\Lambda _0, \boldsymbol{\beta }_0, g_0, \boldsymbol{\gamma }_0, h_0, \zeta _0)$.

Under the Model (2), the cumulative distribution function $F(t|{\textbf{X}},{\textbf{Z}},E)$ can be expressed as:

$$\begin{aligned} F(t|{\textbf{X}},{\textbf{Z}},E)=1-\exp \{-\Lambda _0(t)\exp \{{\boldsymbol{\beta }}_0^\top {\textbf{X}}+g_0({\textbf{Z}})+\{{\boldsymbol{\gamma }}_0^\top {\textbf{X}}+h_0({\textbf{Z}})\}I_{\{E> \zeta _0\}}\}\} . \end{aligned}$$

To guarantee that $F(t|{\textbf{X}},{\textbf{Z}},E)$ is well defined, we require $\Lambda _0(\cdot )$ to be a monotone nondecreasing function satisfying $\Lambda _0(0)=0$; it can be approximated by a monotone spline function $\Lambda _{0,n}(\cdot )=\sum _{j=0}^{q_n}c_jM_j(\cdot |\ell )$, where $c_j\ge 0$ for $j=1,\dots ,q_n$ and $q_n$ indicates the number of basis functions (Ramsay 1988). In this formula, $\Lambda _{0,n}(\cdot )$ is the projection of $\Lambda _0(\cdot )$ onto the finite-dimensional sieve space, where $M_j(\cdot |\ell )$ denotes the integral spline basis function, and $c_j$ represents the corresponding spline coefficient. Assuming that the support set of the observation time *U* lies within the interval $[L_U, R_U]$, where $0< L_U< R_U < \tau$ and $\tau$ is the end time of observation, and all $M_j(\cdot |\ell )$ are nondecreasing piecewise polynomials of degree $\ell$ defined over $[L_U, R_U]$. Let the interval $[L_U, R_U]$ be partitioned as $L_U = t_0< t_1< \dots < t_{m_n+1} = R_U$, where $m_n = O(n^\nu )$ represents the cardinality of the interior nodes with $\nu \in (0, 1/2)$. Given the polynomial degree $\ell$ and the set of interior nodes $\mathcal {S}_n=\{t_1, \dots , t_{m_n}\}$, the total number of basis functions $q_n$ can be determined as $q_n = m_n + \ell$.

In this paper, we use the integrated piecewise polynomial spline function $M_j(\cdot |\ell )=\int _0^t B_j(s|\ell -1)ds$ to estimate the monotonic function $\Lambda _0$. Define

$$\begin{aligned} {\mathcal {F}}:={\mathcal {F}}(\mathcal {S}_n, m_n, \ell )=\left\{ \sum _{j=0}^{q_n}c_jM_j(t|\ell ):\ell \in {\mathbb {N}}, c_j\ge 0 \text { for } j=0,\dots ,q_n,t\in [L_U,R_U] \right\} . \end{aligned}$$

Let ${{\boldsymbol{\eta }}:=(\Lambda , {\boldsymbol{\beta }}, g,{\boldsymbol{\gamma }}, h, \zeta )}$ and denote its true value by ${\boldsymbol{\eta }}_0:=(\Lambda _{0}, {\boldsymbol{\beta }}_0, g_0, {\boldsymbol{\gamma }}_0, h_0, \zeta _0)$. Specifically, we estimate ${\boldsymbol{\eta }}_0$ by

3 $$\begin{aligned} \hat{\boldsymbol{\eta }}_n=(\hat{\Lambda }_n,\hat{\boldsymbol{\beta }}_n,\hat{g}_n,\hat{\boldsymbol{\gamma }}_n,\hat{h}_n,\hat{\zeta }_n)=\arg \max _{{\boldsymbol{\eta }}\in {\mathcal {N}}}\ell _n({\boldsymbol{\eta }}) , \end{aligned}$$

where

$$\begin{aligned} \begin{aligned} \ell _n({\boldsymbol{\eta }})&:=\frac{1}{n}\sum _{i=1}^n\Delta _i\log \left\{ 1-\exp \left\{ -{\Lambda }(U_i)\exp \left\{ {\boldsymbol{\beta }}^\top {\textbf{X}}_i+g({\textbf{Z}}_i) \right. \right. \right. \\&\quad \left. \left. \left. +\{{\boldsymbol{\gamma }}^\top {\textbf{X}}_i+h({\textbf{Z}}_i)\}I_{\{E_i> \zeta \}}\right\} \right\} \right\} \\&\quad -(1-\Delta _i){\Lambda }(U_i)\exp \left\{ {\boldsymbol{\beta }}^\top {\textbf{X}}_i+g({\textbf{Z}}_i)+\{{\boldsymbol{\gamma }}^\top {\textbf{X}}_i+h({\textbf{Z}}_i)\}I_{\{E_i> \zeta \}}\right\} , \end{aligned} \end{aligned}$$

and $${\mathcal {N}}={\mathcal {F}}\times {\mathbb {R}}^p\times {\mathcal {G}}\times {\mathbb {R}}^p\times {\mathcal {G}}\times {\mathbb {R}}$$.

## Asymptotic properties

This section establishes the consistency and asymptotic properties of the estimates in (3). Before discussing them, we first introduce some notation. The estimates of the change point $\zeta _0$ and the remaining parameters $\boldsymbol{\xi }_0=(\Lambda _0,\boldsymbol{\beta }_0,g_0,\boldsymbol{\gamma }_0,h_0)$ are denoted by $\hat{\zeta }_n$ and $\hat{\boldsymbol{\xi }}_n=(\hat{\Lambda }_{n}, \hat{\boldsymbol{\beta }}_n, \hat{g}_n, \hat{\boldsymbol{\gamma }}_n, \hat{h}_n)$, respectively. Note that $\boldsymbol{\eta }=(\boldsymbol{\xi },\zeta )=(\Lambda ,\boldsymbol{\beta },g,\boldsymbol{\gamma },h,\zeta )$. For any *q*-dimensional vector $\boldsymbol{a}=(a_1,\dots ,a_q)^\top \in {\mathbb {R}}^q$, $\Vert \boldsymbol{a}\Vert =\sqrt{\sum _{j=1}^{q}a_j^2}$ and $\Vert \boldsymbol{a}\Vert _\infty =\max _j|a_j|$ denote the $L^2$ norm and $L^\infty$ norm, respectively. For any matrix $W=(w_{ij})\in {\mathbb {R}}^{m\times n}$, let $\Vert W\Vert _{\infty }=\max _{i,j}|w_{ij}|$. For any function *h*(*x*), $\Vert h\Vert _\infty =\sup _x|h(x)|$. Denote $a_n\lesssim b_n$ as $a_n\le cb_n$ and $a_n\gtrsim b_n$ as $a_n\ge cb_n$ for some constant $c>0$ respectively, and $a_n\asymp b_n$ indicates $a_n\lesssim b_n$ and $a_n\gtrsim b_n$. Define the distance of ${\boldsymbol{\eta }}$ as

$$\begin{aligned} \begin{aligned} d({\boldsymbol{\eta }}_1,{\boldsymbol{\eta }}_2)&=\left( \Vert \Lambda _1-\Lambda _2\Vert ^2_{L^2([0,\tau ])}+\Vert {\boldsymbol{\beta }}_1-{\boldsymbol{\beta }}_2\Vert ^2+\Vert g_1-g_2\Vert _{L^2([0,1]^r)}^2\right. \\&\quad +\left. \Vert {\boldsymbol{\gamma }}_1-{\boldsymbol{\gamma }}_2\Vert ^2+\Vert h_1-h_2\Vert _{L^2([0,1]^r)}^2+|\zeta _1-\zeta _2|\right) ^{1/2}, \end{aligned} \end{aligned}$$

where $|\cdot |$ represents the absolute value,

$$\begin{aligned} \Vert g_1-g_2\Vert _{L^2([0,1]^r)}=\left( {\mathbb {E}}\left\{ g_1({\textbf{Z}})-g_2({\textbf{Z}}) \right\} ^2 \right) ^{1/2}, \end{aligned}$$

and

$$\begin{aligned} \Vert \Lambda _1-\Lambda _2\Vert _{L^2([0,\tau ])}=\left( {\mathbb {E}}\left\{ \Lambda _1(U)-\Lambda _2(U) \right\} ^2 \right) ^{1/2}, \end{aligned}$$

respectively. Denote $\hat{\boldsymbol{\theta }}_n=(\hat{\boldsymbol{\beta }}_n^\top ,\hat{\boldsymbol{\gamma }}_n^\top )^\top$, $\tilde{\textbf{X}}(\zeta )=(\textbf{X}^\top ,\textbf{X}^\top I_{\{E>\zeta \}})^\top$, and $\tilde{K}({\textbf{Z}};\zeta )=g({\textbf{Z}})+h({\textbf{Z}})I_{\{E>\zeta \}}$. Write

$$\begin{aligned} {\boldsymbol{h}}_{{\boldsymbol{\eta }}}(\boldsymbol{V})=\exp \left\{ \log {\Lambda }(U)+{\boldsymbol{\beta }}^\top {\textbf{X}}+g({\textbf{Z}})+\{{\boldsymbol{\gamma }}^\top {\textbf{X}}+h({\textbf{Z}})\}I_{\{E> \zeta \}} \right\} , \end{aligned}$$

where $\boldsymbol{V}=(U,\textbf{X},\textbf{Z},E)$, and define

$$\begin{aligned} m_{{\boldsymbol{\eta }}}(\boldsymbol{V})=\Delta \log (1-\exp \{-{\boldsymbol{h}}_{{\boldsymbol{\eta }}}(\boldsymbol{V})\})-(1-\Delta ){\boldsymbol{h}}_{{\boldsymbol{\eta }}}(\boldsymbol{V}), \end{aligned}$$

then $\ell _n({\boldsymbol{\eta }})={\mathbb {P}}_nm_{{\boldsymbol{\eta }}}(\boldsymbol{V}):=\frac{1}{n}\sum _{i=1}^n m_{{\boldsymbol{\eta }}}(\boldsymbol{V}_i)$ and define $\ell ({\boldsymbol{\eta }})={\mathbb {P}} m_{{\boldsymbol{\eta }}}(\boldsymbol{V}):=\int m_{{\boldsymbol{\eta }}}(\boldsymbol{V}) dP$.

To establish the theoretical results, the following regularity conditions are required:

1. $$K=O(\log n)$$, $$n\alpha _n^2\lesssim \min (p_k)_{k=1,\dots ,K}\le \max (p_k)_{k=1,\dots , K}\lesssim n$$ and $$s=O(n\alpha _n^2\log n)$$.
2. For $\nu \in (0,\frac{1}{2})$, assume the maximum spacing of the knots is $O(n^{-\nu })$, i.e.
  $$\begin{aligned} \max _{1\le k\le m_n+1}|t_k-t_{k-1}|=O(n^{-\nu }). \end{aligned}$$
3. The true parameter vector ${\boldsymbol{\theta }}_0$ lies in the interior of a compact subset of ${\mathbb {R}}^{2p}$.
4. The *u*th derivative of $\Lambda _0$ satisfies the Lipschitz conditions with $u \ge 1$ and $\Lambda _0$ increases in $[0,\tau ]$.
5. The support of *U* is an interval within $[L_U,R_U]$, with $0<L_U<R_U<\tau$.
6. Assume the covariates $({\textbf{X}},{\textbf{Z}})$ lie in a bounded subset of ${\mathbb {R}}^{p+r}$. Without loss of generality, we further assume ${\textbf{Z}}\in [0,1]^r$.
7. ${\boldsymbol{h}}_{{\boldsymbol{\eta }}}(\boldsymbol{V})$ is almost surely bounded away from 0 for all ${\boldsymbol{\eta }}\in \{{\boldsymbol{\eta }}:d({\boldsymbol{\eta }},{\boldsymbol{\eta }}_0)<\epsilon \}$ with some small $\epsilon >0$.
8. For any ${\textbf{Z}}\in [0,1]^r$, assume that ${\mathbb {E}} g_0({\textbf{Z}})=0$ and ${\mathbb {E}} h_0({\textbf{Z}})=0$.
9. We assume the existence of a change point $\zeta _0\in {\mathbb {R}}$, i.e. ${\mathbb {P}}({\boldsymbol{\gamma }}_0^\top {\textbf{X}}+h_0({\textbf{Z}})\ne 0)>0$. Also, for any ${\boldsymbol{\theta }}\ne {\boldsymbol{\theta }}_0$ and fixed $\zeta \in {\mathbb {R}}$, ${\mathbb {P}}({\boldsymbol{\theta }}^\top \tilde{\textbf{X}}(\zeta )\ne {\boldsymbol{\theta }}_0^\top \tilde{\textbf{X}}(\zeta ))>0.$
10. The variable *E* admits a density $h_3(\cdot )$ that is strictly positive, bounded, and continuous on a neighborhood of $\zeta _0$.
11. The information matrix $I({\boldsymbol{\theta }}_0;\zeta _0)$ defined in Theorem 4 is nonsingular.

Condition (C1) determines the structure and complexity of the deep neural network. Condition (C2) is a mild regularity assumption on the knot placement and is similar to the conditions in Ramsay (1988). Condition (C3) is a standard assumption in semiparametric estimation The smoothness and monotonicity conditions on $\Lambda _0(\cdot )$ in (C4) guarantee that it can be well approximated by a monotone spline basis. Conditions (C5)-(C7) are needed for the calculation in the proofs of Theorems 1–5. Conditions (C8) and (C9) are required to ensure that the model parameters are identifiable. Condition (C10) indicates that *E* is continuously distributed in a bounded neighborhood of $\zeta _0$, which is crucial for establishing the asymptotic independence and asymptotic properties at $\zeta _0$. (C11) ensures the asymptotic efficiency of the regression parameter ${\boldsymbol{\theta }}_0$.

### Theorem 1

(Identifiability) Under the conditions (C1)-(C9), the parameter $\boldsymbol{\eta }=(\boldsymbol{\xi },\zeta )$ is identifiable.

Theorem 1 limits the range of the change point, illustrating that this methodology is identifiable only when $\zeta _0\in (\min _i(E_i),\max _i(E_i))$, i.e. ${\mathbb {P}}(\{E>\zeta _0\})\in (0,1)$. Another characteristic of identifiability is the event that ${\boldsymbol{\gamma }}_0\ne {\boldsymbol{0}}$ or $g_0(\cdot )\ne 0$ occurs.

Before deriving the overall convergence rate, we introduce a Hölder class that contains both $g_0$ and $h_0$. Let $\kappa$ and *B* be two positive constants, and $\lfloor \kappa \rfloor$ is the largest integer smaller than $\kappa$. A $(\kappa ,{{B}})$-Hölder class of smooth functions is denoted as

$$\begin{aligned} \begin{aligned} {\mathcal {H}}_{r}^{\kappa }({\mathbb {D}},{{{B}}})&=\left\{ g:{\mathbb {D}}\subset {\mathbb {R}}^r\rightarrow {\mathbb {R}}: \sum _{{\boldsymbol{\upsilon }}: |{\boldsymbol{\upsilon }}|<{{\kappa }}}\Vert \partial ^{{\boldsymbol{\upsilon }}} g\Vert _\infty \right. \\&\quad \left. +\sum _{{\boldsymbol{\upsilon }}:|{\boldsymbol{\upsilon }}|=\lfloor \kappa \rfloor }\sup _{x,y\in {\mathbb {D}},x\ne y}\frac{|\partial ^{{\boldsymbol{\upsilon }}} g(x)-\partial ^{{\boldsymbol{\upsilon }}} g(y)|}{\Vert x-y\Vert _\infty ^{\kappa -[\kappa ]}}\le {{{B}}} \right\} , \end{aligned} \end{aligned}$$

where $\partial ^{{\boldsymbol{\upsilon }}}=\partial ^{\upsilon _1}\cdots \partial ^{\upsilon _r}$ with ${\boldsymbol{\upsilon }}=(\upsilon _1,\dots ,\upsilon _r)^\top$ and $|{\boldsymbol{\upsilon }}|=\sum _{j=1}^r\upsilon _j$. Denote $K>0$, $R\in {\mathbb {N}}^+$, ${\boldsymbol{\kappa }}=(\kappa _0,\dots ,\kappa _{K})^\top \in {\mathbb {R}}_+^{K+1}$, and let ${\boldsymbol{d}}=(d_0,\dots ,d_{K+1})\in {\mathbb {N}}_+^{K+2}$, $\tilde{{\boldsymbol{d}}}=(\tilde{d}_0,\dots ,\tilde{d}_{K})^\top \in {\mathbb {N}}_+^{K+1}$ with $\tilde{d}_j\le d_j$ where $j=0,\dots ,L$. Further, assume that the functions $g_0$ and $h_0$ belong to a composite smoothness function class:

$$\begin{aligned} \begin{aligned} {\mathcal {H}}(K,{\boldsymbol{\kappa }},{\boldsymbol{d}},\tilde{\boldsymbol{d}},{{{B}}})&:=\left\{ h=h_K\circ \cdots \circ h_0: h_i=(h_{i1},\dots ,h_{id_{i+1}})^\top \text { and} \right. \\&\quad \left. h_{ij}\in {\mathcal {H}}_{\tilde{d}_i}^{\kappa _i}\left( [a_i,b_i]^{\tilde{d}_i},{B}\right) \text { for some }|a_i|,|b_i|\le {{{B}}} \right\} , \end{aligned} \end{aligned}$$

where $\tilde{\boldsymbol{d}}$ denotes the intrinsic dimension of the function in this class. Furthermore, denote $\tilde{\upsilon }_i=\upsilon _i\prod _{j=i+1}^K(\upsilon _j\wedge 1)$ and $\alpha _n=\max _{i=0,\dots ,K}n^{-{\tilde{\upsilon }_i}/{(2\tilde{\upsilon }_i+\tilde{d}_i)}}$, where $a\wedge b=\min \{a,b\}$.

### Theorem 2

(Consistency and convergence rate) Let $(2u+1)^{-1}<\nu <(2u)^{-1}$ with $u\ge 1$ and $q_n=O(n^\nu )$. Suppose conditions (C1)-(C9) hold, we have $d(\hat{\boldsymbol{\eta }}_n,{\boldsymbol{\eta }}_0)=O_P(\alpha _n\log ^2 n+n^{-u\nu })$.

Theorem 2 illustrates that the rate is determined by the smoothness $\alpha _n$, the intrinsic dimension $\tilde{\boldsymbol{d}}$ of the DNN, the smoothness $\ell$, and the maximum number of nodes $m_n$ of the spline function. As a result, when the intrinsic dimension $\tilde{\boldsymbol{d}}$ is relatively small, the suggested strategy can mitigate the curse of dimensionality and has a faster convergence rate. It is well known that when nonparametric smoothing techniques (such as splines or kernel functions) are used to estimate $g_0$, the model suffers from a severe “curse of dimensionality”. A key advantage of the DNN estimator over traditional smoothing methods is its ability to alleviate the curse of dimensionality by accurately projecting data into a lower-dimensional representation space (Anthony and Bartlett 1999).

We next establish the semiparametric efficiency. Unlike the classical regression model, the unknown change point affects the procedure of estimation and projection, which makes it difficult to obtain the score and information functions (Pons 2003; Deng et al. 2022). Therefore, before establishing the semiparametric efficiency, we first show that the effect of the change point is asymptotically negligible; in other words, when *n* is large enough, $\hat{\zeta }_n$ is asymptotically independent of $\hat{\boldsymbol{\xi }}_n$. In particular, denote two counting processes

$$ \begin{array}{l} N_{1i}(t) = (1-\Delta _i) I(U_i \le t ),\\ N_{2i}(t) = \Delta _i I(U_i \le t ). \end{array} $$

For simplicity, let

$$ \begin{array}{l} {\boldsymbol{m}}(t;{\boldsymbol{\xi }},\zeta ) = -{\Lambda }(t)\exp \{{\boldsymbol{\beta }}^\top {\textbf{X}}+g({\textbf{Z}})+\{{\boldsymbol{\gamma }}^\top {\textbf{X}}+h({\textbf{Z}})\}I_{\{E>\zeta \}}\},\\ {\mathcal {M}}(t;{\boldsymbol{\xi }}) = {\Lambda }(t)\exp \{{\boldsymbol{\beta }}^\top {\textbf{X}}+g({\textbf{Z}})\}\left[ \exp \{{\boldsymbol{\gamma }}^\top {\textbf{X}}+h({\textbf{Z}}) \}-1\right] ,\\ \end{array} $$

$$\begin{aligned} {\mathcal {M}}^+(t;{\boldsymbol{\xi }})=\log \left( \frac{1-\exp \left\{ -{\Lambda }(t)\exp \{{\boldsymbol{\beta }}^\top {\textbf{X}}+ g({\textbf{Z}})+\{{\boldsymbol{\gamma }}^\top {\textbf{X}}+ h({\textbf{Z}})\}(I_{\{E>\zeta _0 \}}-1) \} \right\} }{1-\exp \left\{ -{\Lambda }(t)\exp \{{\boldsymbol{\beta }}^\top {\textbf{X}}+ g({\textbf{Z}})+\{{\boldsymbol{\gamma }}^\top {\textbf{X}}+ h({\textbf{Z}})\}I_{\{E>\zeta _0 \}} \} \right\} } \right) , \end{aligned}$$

$$\begin{aligned} {\mathcal {M}}^-(t;{\boldsymbol{\xi }})=\log \left( \frac{1-\exp \left\{ -{\Lambda }(t)\exp \{{\boldsymbol{\beta }}^\top {\textbf{X}}+ g({\textbf{Z}})+\{{\boldsymbol{\gamma }}^\top {\textbf{X}}+ h({\textbf{Z}})\}(I_{\{E>\zeta _0 \}}+1) \} \right\} }{1-\exp \left\{ -{\Lambda }(t)\exp \{{\boldsymbol{\beta }}^\top {\textbf{X}}+ g({\textbf{Z}})+\{{\boldsymbol{\gamma }}^\top {\textbf{X}}+ h({\textbf{Z}})\}I_{\{E>\zeta _0 \}} \} \right\} } \right) , \end{aligned}$$

and

$$\begin{aligned} \begin{aligned} Q_n({\boldsymbol{\xi }}_0,\hat{\zeta }_n)&=\sum _{i=1}^n(1-\Delta _i){\mathcal {M}}(U_i;{\boldsymbol{\xi }}_0)\tilde{I}(\hat{\zeta }_n)\\&\quad +\Delta _i\left\{ {\mathcal {M}}^+(U_i;{\boldsymbol{\xi }}_0)I_{\{\zeta _0<E\le \hat{\zeta }_n\}}-{\mathcal {M}}^-(U_i;{\boldsymbol{\xi }}_0)I_{\{\hat{\zeta }_n<E\le \zeta _0\}} \right\} . \end{aligned} \end{aligned}$$

### Theorem 3

(Asymptotic independence) Under (C1)-(C10), there exists

$$\begin{aligned} {\ell _n(\hat{\boldsymbol{\eta }}_n)}=n^{-1}Q_n({\boldsymbol{\xi }}_0,\hat{\zeta }_n)+\ell _n(\hat{\boldsymbol{\xi }}_n,\zeta _0)+o_P(1), \end{aligned}$$

where

$$\begin{aligned} \begin{aligned} \ell _n(\hat{\boldsymbol{\xi }}_n,\zeta _0)&:=\frac{1}{n}\sum _{i=1}^n\Delta _i\log \left\{ 1-\exp \left\{ -\Lambda (U_i)\exp \left\{ \boldsymbol{\beta }^\top \textbf{X}_i+g(\textbf{Z}_i)+\{\boldsymbol{\gamma }^\top \textbf{X}_i+h(\textbf{Z}_i)\}I_{\{E_i> \zeta _0\}}\right\} \right\} \right\} \\&\quad -(1-\Delta _i)\Lambda (U_i)\exp \left\{ \boldsymbol{\beta }^\top \textbf{X}_i+g(\textbf{Z}_i)+\{\boldsymbol{\gamma }^\top \textbf{X}_i+h(\textbf{Z}_i)\}I_{\{E_i> \zeta _0\}}\right\} . \end{aligned} \end{aligned}$$

Furthermore, $\hat{\boldsymbol{\xi }}_n$ is asymptotically independent of $\hat{\zeta }_n$.

The unknown change point causes inconvenience in statistical inference, especially in deriving the asymptotic distribution. Based on asymptotic independence, we establish the effective score and information bound for $\theta _{0}$.

Let $$ \Omega _{0} = \left\{ \begin{gathered} \Lambda \in L^{2} \left( {\left[ {0,\tau } \right]} \right) \hfill \\ :\Lambda (\tau ) < \infty \hfill \\ \end{gathered} \right\} $$, and let $$ \begin{gathered} {\mathcal{H}}_{0} = \{ g \in {\mathcal{H}}\{ K,\kappa ,d,\tilde{d},B\} \hfill \\ \quad \quad :{\mathbb{E}}\{ g(Z)\} = 0\} \hfill \\ \end{gathered}$$. Denote $\Omega _{\Lambda _0}$ be the collection of all subfamilies $\{\log \Lambda _t: t\in (-1,1)\}\subset \{\log \Lambda :\Lambda \in \Omega _0\}$ such that $\lim _{t\rightarrow \infty }\Vert t^{-1}(\log \Lambda _t-\log \Lambda _0)-a\Vert _{L^2([0,\tau ])}=0$ where $a\in L^2([0,\tau ])$, and let

$$\begin{aligned} \begin{aligned} {\mathbb {T}}_{\Lambda _0}&=\left\{ a\in L^2([0,\tau ]): \lim _{t\rightarrow \infty }\Vert t^{-1}(\log \Lambda _t-\log \Lambda _0)-a\Vert _{L^2([0,\tau ])}=0 \right. \\&\quad \left. \text { for some subfamily }\{\log \Lambda _t: t\in (-1,1)\}\subset \Omega _0 \right\} . \end{aligned} \end{aligned}$$

Similarly, define

$$\begin{aligned} \begin{aligned} {\mathbb {T}}_{\tilde{K}_0}&=\left\{ b\in L^2([0,1]^r): \lim _{s\rightarrow \infty }\Vert s^{-1}(\tilde{K}_s-\tilde{K}_0)-b\Vert _{L^2([0,1]^r)}=0 \right. \\&\quad \left. \text { for some subfamily }\{\tilde{K}_s: s\in (-1,1)\}\subset {\mathcal {H}}_{\tilde{K}_0},\text { and }{\mathbb {E}}\{b({\textbf{Z}})\}=0 \right\} , \end{aligned} \end{aligned}$$

where $\mathcal {H}_{\tilde{K}_0}$ denotes the collection of all subfamilies of $\{\tilde{K}_s(\cdot ;\zeta _0)\in L^2([0,1]^r):s\in (-1,1) \}\subset \mathcal {H}_0$ such that $\lim _{s\rightarrow 0}\Vert u^{-1}(\tilde{K}_s-K_0)-b\Vert =0$ and $b\in L^2([0,1]^r)$. Let $\bar{\mathbb {T}}_{\Lambda _0}$ and $\bar{\mathbb {T}}_{\tilde{K}_0}$ be the linear span of ${\mathbb {T}}_{\Lambda _0}$ and ${\mathbb {T}}_{\tilde{K}_0}$, respectively. Denote the single observation of the log-likelihood $\ell ({\boldsymbol{\eta }}|{\textbf{Y}})=\Delta \log (1-\exp \{-{\boldsymbol{h}}_{{\boldsymbol{\eta }}}(\boldsymbol{V})\})-(1-\Delta ){\boldsymbol{h}}_{{\boldsymbol{\eta }}}(\boldsymbol{V})$, where $\textbf{Y}=(\Delta ,\boldsymbol{V})$. The score function w.r.t. ${\boldsymbol{\theta }}$ is

$$\begin{aligned} \dot{\ell }_{\boldsymbol{\theta }}({\boldsymbol{\eta }}_0)=\tilde{{\textbf{X}}}(\zeta _0){\mathcal {Q}}({\textbf{Y}};{\boldsymbol{\xi }}_0,\zeta _0), \end{aligned}$$

where

$$\begin{aligned} {\mathcal {Q}}({\textbf{Y}};{\boldsymbol{\xi }},\zeta )={\boldsymbol{h}}_{{\boldsymbol{\eta }}}(\boldsymbol{V})\left\{ \Delta \frac{\exp \{-{\boldsymbol{h}}_{{\boldsymbol{\eta }}}(\boldsymbol{V}) \}}{1-\exp \{-{\boldsymbol{h}}_{{\boldsymbol{\eta }}}(\boldsymbol{V}) \}}-(1-\Delta ) \right\} . \end{aligned}$$

Let a parametric smooth model $(\Lambda _t(U),\tilde{K}_s({\textbf{Z}};\zeta _0))$ satisfy $\Lambda _t(U)|_{t=0}=\Lambda _0(U)$ and $\tilde{K}_s({\textbf{Z}};\zeta _0)|_{s=0}=\tilde{K}_0({\textbf{Z}};\zeta _0)$, with $\left. \frac{\partial }{\partial t}\Lambda _t(U)\right| _{t=0}=a(U)$ and $\left. \frac{\partial }{\partial s}\tilde{K}_s({\textbf{Z}};\zeta _0)\right| _{s=0}=b({\textbf{Z}})$, the score functions for $\Lambda _0$ and $\tilde{K}_0(\cdot ;\zeta _0)$ are

$$\begin{aligned} \dot{\ell }_{\Lambda }({{\boldsymbol{\eta }}_0})[a]=\frac{\partial \ell ({\boldsymbol{\theta }}_0, \Lambda _t, \tilde{K}_0\mid {\textbf{Y}})}{\partial t}\Big |_{t=0}=a(U){\mathcal {Q}}({\textbf{Y}};{\boldsymbol{\xi }}_0,\zeta _0) \end{aligned}$$

and

$$\begin{aligned} \dot{\ell }_{\tilde{K}}({{\boldsymbol{\eta }}_0})[b]=\frac{\partial \ell ({\boldsymbol{\theta }}_0, \Lambda _0, \tilde{K}_s\mid {\textbf{Y}})}{\partial s}\Big |_{s=0}=b({\textbf{Z}}){\mathcal {Q}}({\textbf{Y}};{\boldsymbol{\xi }}_0,\zeta _0), \end{aligned}$$

respectively. For ${\boldsymbol{a}}=(a_1,\dots ,a_{2p})^\top \in \bar{\mathbb {T}}_{\Lambda _0}$, let $\dot{\ell }_{\Lambda }({{\boldsymbol{\eta }}_0})[{\boldsymbol{a}}]=(\dot{\ell }_{\Lambda }({{\boldsymbol{\eta }}_0})[a_1],\dots ,\dot{\ell }_{\Lambda }({{\boldsymbol{\eta }}_0})[a_{2p}])^\top$, and for ${\boldsymbol{b}}=(b_1,\dots ,b_{2p})^\top \in \bar{\mathbb {T}}_{\tilde{K}_0}$, define $\dot{\ell }_{\tilde{K}}({{\boldsymbol{\eta }}_0})[{\boldsymbol{b}}]=(\dot{\ell }_{\tilde{K}}({{\boldsymbol{\eta }}_0})[b_1],\dots ,\dot{\ell }_{\tilde{K}}({{\boldsymbol{\eta }}_0})[b_{2p}])^\top$. The score and information bound are given in the following theorem.

### Theorem 4

Under (C1)–(C11),

if $({\boldsymbol{a}}_*^\top ,{\boldsymbol{b}}_*^\top )^\top \in \bar{\mathbb {T}}_{\Lambda _0}\times \bar{\mathbb {T}}_{\tilde{K}_0}$ minimizes the distance

$$\begin{aligned} {\mathbb {E}}\left\{ {\mathcal {Q}}^2({\textbf{Y}};{\boldsymbol{\xi }}_0,\zeta _0)[(\tilde{\textbf{X}}(\zeta )-{\boldsymbol{a}}(U)-{\boldsymbol{b}}({\textbf{Z}}))\odot (\tilde{\textbf{X}}(\zeta )-{\boldsymbol{a}}(U)-{\boldsymbol{b}}({\textbf{Z}}))] \right\} , \end{aligned}$$

where ${\boldsymbol{y}}\odot {\boldsymbol{y}}=(y_1^2,\dots ,y_q^2)^\top$ for any *q*-dimensional vector ${\boldsymbol{y}}=(y_1,\dots ,y_q)^\top \in {\mathbb {R}}^q$ and the minimization operates component-wise on the vector, the efficient score function for ${\boldsymbol{\theta }}_0$ is

$$\begin{aligned} U({\boldsymbol{\theta }}_0;\zeta _0)=\ell _{\boldsymbol{\theta }}^*({\textbf{Y}},{\boldsymbol{\eta }}_0)=\{\tilde{\textbf{X}}(\zeta _0)-{\boldsymbol{a}}_*(U)-{\boldsymbol{b}}_*({\textbf{Z}}) \}{\mathcal {Q}}({\textbf{Y}};{\boldsymbol{\xi }}_0,\zeta _0), \end{aligned}$$

and the information matrix

$$\begin{aligned} I({\boldsymbol{\theta }}_0;\zeta _0)={\mathbb {E}}(U({\boldsymbol{\theta }}_0;\zeta _0)^{\otimes 2})={\mathbb {E}}\left\{ {\mathcal {Q}}^2({\textbf{Y}};{\boldsymbol{\xi }}_0,\zeta _0)\{\tilde{\textbf{X}}(\zeta _0)-{\boldsymbol{a}}_*(U)-{\boldsymbol{b}}_*({\textbf{Z}}) \}^{\otimes 2} \right\} . \end{aligned}$$

After determining the score function and the information bound of ${\boldsymbol{\theta }}$, we present the asymptotic distribution of ${\hat{\boldsymbol{\theta }}_n}$ and $\hat{\zeta }_n$. Denote two variables

$$\begin{aligned} \begin{aligned} \xi ^+=\xi ^+(U,\Delta ,\textbf{X},\textbf{Z},E) = (1-\Delta ) {\mathcal {M}}(t,{\boldsymbol{\xi }}_0)+\Delta {\mathcal {M}}^+(t,{\boldsymbol{\xi }}_0), \end{aligned} \end{aligned}$$

$$\begin{aligned} \begin{aligned} \xi ^-=\xi ^-(U,\Delta ,\textbf{X},\textbf{Z},E) = (1-\Delta ) {\mathcal {M}}(t,{\boldsymbol{\xi }}_0)+\Delta {\mathcal {M}}^-(t,{\boldsymbol{\xi }}_0), \end{aligned} \end{aligned}$$

respectively, and define

$$\begin{aligned} \tilde{Q}^+_n(v)=I_{\{v>0\}}\sum _{i=1}^n\xi _i^+I_{\{\zeta _0<E_i\le \zeta _0+n^{-1}v\}}, \end{aligned}$$

$$\begin{aligned} \tilde{Q}^-_n(v)=I_{\{v<0\}}\sum _{i=1}^n\xi _i^-I_{\{\zeta _0+n^{-1}v<E_i\le \zeta _0\}}, \end{aligned}$$

where $\xi _i^+=\xi ^+(U_i,\Delta _i,{\textbf{X}}_i,{\textbf{Z}}_i,E_i)$ and $\xi _i^-=\xi ^-(U_i,\Delta _i,{\textbf{X}}_i,{\textbf{Z}}_i,E_i)$. Furthermore, for $v\in {\mathbb {R}}^+$, define

$$\begin{aligned} \tilde{Q}^+(v)=I_{\{v>0\}}\sum _{j\ge 1}\frac{e^{-vh_3(\zeta _0)}(vh_3(\zeta _0))^j}{(j-1)!}{\mathbb {E}}(\xi ^+|E=\zeta _0^+)\;, \end{aligned}$$

where

$$\begin{aligned} \begin{aligned} {\mathbb {E}}(\xi ^+\mid E=\zeta _0^+)&={\mathbb {E}}\left\{ (1-\Delta ) {\mathcal {M}}(U,{\boldsymbol{\xi }}_0)\right. \\&\quad +\left. \Delta \log \left( \frac{1-\exp \{-\Lambda _0(U)\exp \{{\boldsymbol{\beta }}_0^\top {\textbf{X}}+g_0({\textbf{Z}})\}}{1-\exp \{-\Lambda _0(U)\exp \{({\boldsymbol{\beta }}_0+{\boldsymbol{\gamma }}_0)^\top {\textbf{X}}+g_0({\textbf{Z}})+h_0({\textbf{Z}})\}} \right) \right\} , \end{aligned} \end{aligned}$$

and for $v\in {\mathbb {R}}^-$, define

$$\begin{aligned} \tilde{Q}^-(v)=I_{\{v<0\}}\sum _{j\ge 1}\frac{e^{vh_3(\zeta _0)}(-vh_3(\zeta _0))^j}{(j-1)!}{\mathbb {E}}(\xi ^-|E=\zeta _0)\; , \end{aligned}$$

where

$$\begin{aligned} \begin{aligned} {\mathbb {E}}(\xi ^-\mid E=\zeta _0)&={\mathbb {E}}\left\{ (1-\Delta ) {\mathcal {M}}(U,{\boldsymbol{\xi }}_0)\right. \\&\quad -\left. \Delta \log \left( \frac{1-\exp \{-\Lambda _0(U)\exp \{{\boldsymbol{\beta }}_0^\top {\textbf{X}}+g_0({\textbf{Z}})\}}{1-\exp \{-\Lambda _0(U)\exp \{({\boldsymbol{\beta }}_0+{\boldsymbol{\gamma }}_0)^\top {\textbf{X}}+g_0({\textbf{Z}})+h_0({\textbf{Z}})\}} \right) \right\} . \end{aligned} \end{aligned}$$

### Theorem 5

(Semiparametric efficiency) Under (C1)-(C11),

if $n\alpha _n^4\rightarrow 0$ as $n\rightarrow \infty$ and $(2u+1)^{-1}<\nu <(2u)^{-1}$ for $u\ge 1$, the parametric regression parameter estimator $\hat{\boldsymbol{\theta }}_n=(\hat{\boldsymbol{\beta }}^\top _n,\hat{\boldsymbol{\gamma }}^\top _n)^\top$ satisfies $\sqrt{n}(\hat{\boldsymbol{\theta }}_n-{\boldsymbol{\theta }}_0){\mathop {\rightarrow }\limits ^{d}}N({\boldsymbol{0}},I^{-1}({\boldsymbol{\theta }}_0;\zeta _0))$

where $I({\boldsymbol{\theta }}_0;\zeta _0)$ is defined in Theorem 4. The change point estimator $\hat{\zeta }_n$ satisfies

$$\begin{aligned} n(\hat{\zeta }_n-\zeta _0){\mathop {\rightarrow }\limits ^{d}}\inf \left\{ v:v=\arg \max _u \tilde{Q}(v)\right\} , \end{aligned}$$

where $$\tilde{Q}(v):=\tilde{Q}^+(v)-\tilde{Q}^-(v)$$.

Theorem 5 presents the $\sqrt{n}$-consistency and asymptotic normality of the estimate of the regression parameter and shows that the estimate of the change point converges distributionally to a distribution of the maximum value of a composite Poisson-type process with rate $n^{-1}$.

## Model diagnostic

In practice, the detection of change points is a crucial problem. Accurate identification improves understanding of the data structure, enhances predictive models, and supports better decision-making. We aim to test whether a change point exists, i.e., the null hypothesis $H_0:{\boldsymbol{\gamma }}_0={\boldsymbol{0}},~ h_0(\cdot )=0.$ Note that if ${\boldsymbol{\gamma }}^\top {\textbf{X}}+h({\textbf{Z}})$ is degenerate (identically zero) or the threshold diverges ($\zeta _0=\pm \infty$), the change-point model fails to be identifiable. If $H_{0}$ holds, model (2) reduces to the deep partially linear Cox model (1) and one can estimate ${\boldsymbol{\beta }}_0$, $\Lambda _0(\cdot )$ and $g_0(\cdot )$ by the estimation procedure proposed by Wu et al. (2024). To test $H_0$, we propose a functional SUP statistic inspired by the supremum test (SUP) proposed by Kosorok and Song (2007).

Denote $\boldsymbol{L}_n(\boldsymbol{\gamma },h,\Lambda ,\boldsymbol{\beta },g,\zeta )=\sum _{i=1}^n\Delta _i\log (1-\exp \{-\boldsymbol{h}_{\boldsymbol{\eta }}(\boldsymbol{V}_i)\})-(1-\Delta _i)\boldsymbol{h}_{\boldsymbol{\eta }}(\boldsymbol{V}_i)$. The score functional evaluated at direction $f\in \mathcal {G}$ is defined as

$$\begin{aligned} \begin{aligned} {\boldsymbol{U}}_n(\zeta ,f,{\boldsymbol{\xi }})&= \frac{\partial }{\partial (\boldsymbol{\gamma }^\top ,\varepsilon )^\top }\boldsymbol{L}_n(\boldsymbol{\gamma },h+\varepsilon f,\Lambda ,\boldsymbol{\beta },g,\zeta )\bigg |_{\varepsilon =0}\\&=\sum _{i=1}^n \mathcal {Q}(\textbf{Y}_i;\boldsymbol{\xi },\zeta )\frac{\partial }{\partial (\boldsymbol{\gamma }^\top ,\varepsilon )^\top }\left\{ \log \Lambda (U_i)+\boldsymbol{\beta }^\top \textbf{X}_i+g(\textbf{Z}_i)\right. \\&\quad \left. +\{\boldsymbol{\gamma }^\top \textbf{X}_i+h(\textbf{Z}_i)+\varepsilon f(\textbf{Z}_i)\}I_{\{E_i>\zeta \}}\right\} \bigg |_{\varepsilon =0} \\&=\sum _{i=1}^nI_{\{E_i>\zeta \}}{\mathcal {Q}}({\textbf{Y}}_i;{\boldsymbol{\xi }},\zeta )(\textbf{X}_i^\top ,f(\textbf{Z}_i))^\top , \end{aligned} \end{aligned}$$

and the covariance of $\boldsymbol{U}_n(\zeta ,f,\boldsymbol{\xi })$ is

$$\begin{aligned} {\boldsymbol{\Sigma }}_n(\zeta ,f,{\boldsymbol{\xi }}) =\sum _{i=1}^nI_{\{E_i>{\zeta }\} }{\mathcal {Q}}^2({\textbf{Y}}_i;{\boldsymbol{\xi }},\zeta )(\textbf{X}_i^\top ,f(\textbf{Z}_i))^\top (\textbf{X}_i^\top ,f(\textbf{Z}_i)). \end{aligned}$$

Then the SUP statistic is defined as

$$ \textrm{SUP}=\sup _{\zeta \in [\zeta _1,\zeta _2]} \sup _{f\in {\mathcal {G}}}{\boldsymbol{U}_n}(\zeta ,f,\tilde{\boldsymbol{\Omega }}_n)^\top {\boldsymbol{\Sigma }_n}(\zeta ,f,\tilde{\boldsymbol{\Omega }}_n)^{-1} {\boldsymbol{U}_n}(\zeta ,f,\tilde{\boldsymbol{\Omega }}_n), $$

where $\tilde{\boldsymbol{\Omega }}_n=(\tilde{\Lambda }_n,\tilde{\boldsymbol{\beta }}_n,\tilde{g}_n,\boldsymbol{0},0)$, and where $\tilde{\Lambda }_n$, $\tilde{\boldsymbol{\beta }}_n$, and $\tilde{g}_n$ are the restricted MLE of $\boldsymbol{\beta }_0$, $\Lambda _0$ and $g_0$ under $H_0$, respectively.

For application, the change point $\zeta$ can be selected by grid search, and the function *f* can be chosen from the DNN function space $\mathcal {G}$ to match the potentially complex nonlinear forms of the direction. However, directly selecting $f \in \mathcal {G}$ may be infeasible due to the high dimensionality and complexity of the DNN function space. In practice, one can instead work with a simpler space that contains G, such as a finite-dimensional linear span $\mathcal {T}$, and choose *f* from $\mathcal {T}$. To reduce the computational burden of evaluating *f*(*Z*), the selection of direction functions can be further decomposed into a sequence of one-dimensional problems. Specifically, for each dimension $j=1,\dots ,r$ of $\textbf{Z}$, we can select *f* from ${\mathcal {T}}_{j}$, the projection on the *j*th dimension of $\mathcal {T}$, and calculate the corresponding score-based statistics. Ultimately, we take the maximum of the statistics across all dimensions, which substantially reduces computational complexity while retaining flexibility. For example, if we take ${\mathcal {T}} = \text {span}\{I_{\{Z_{1}<z_1,\dots ,Z_r<z_r\}}\}$ to replace $\mathcal {G}$, the projection on its *j*th dimension ${\mathcal {T}}_{j}=\text {span}\{I_{\{Z_j<z_j\}}\}$ can be used to choose *f*; we can take $\textbf{Z}=_{\{Z_j<z_j\}}\in \mathcal {T}_j$ for $j=1,\dots ,r$ and then take the supremum over *j*, rather than using $\textbf{Z}=I_{\{Z_1<z_1,\dots ,Z_r<z_r\}}\in \mathcal {T}$ to compute the SUP statistic directly. In summary, the $\textrm{SUP}_k$ statistic is defined as

$$\begin{aligned} \textrm{SUP}_k=\sup _{\zeta \in \{\zeta _1^*,\dots ,\zeta _k^*\}}\sup _{j\in \{1,\dots ,r\}} \sup _{f\in {\mathcal {T}}_{j}}{\boldsymbol{U}_n}(\zeta ,f,\tilde{\boldsymbol{\Omega }}_n)^\top {\boldsymbol{\Sigma }_n}(\zeta ,f,\tilde{\boldsymbol{\Omega }}_n)^{-1} {\boldsymbol{U}_n}(\zeta ,f,\tilde{\boldsymbol{\Omega }}_n), \end{aligned}$$

where $\{\zeta _1^*,\dots ,\zeta _k^*\} \subset [\zeta _1,\zeta _2]$. We construct the permutation distribution of $\textrm{SUP}_{k}$ by repeatedly permuting E and recomputing the statistic. At a significance level $\alpha$, reject $H_0$ if $\textrm{SUP}_{k}$ exceeds the upper $\alpha$-quantile of its permutation distribution; otherwise, do not reject $H_0$. The algorithm below summarizes the testing procedure.

**Algorithm 1:**
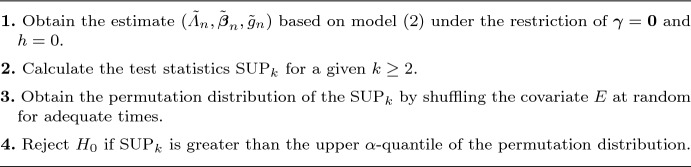
Test procedure for $H_0$

## Simulation studies

In this section, we first present the computational procedure. The loss is the negative log-likelihood function, which is nonconcave in the parameters ${\boldsymbol{\beta }}$, ${\boldsymbol{\gamma }}$, $W_j$, ${\boldsymbol{\nu }}_j$, and $c_j$; consequently, conventional optimization methods may perform poorly. To overcome this difficulty, we propose an iterative profiling estimation procedure, detailed in the algorithm below.

**Algorithm 2:**
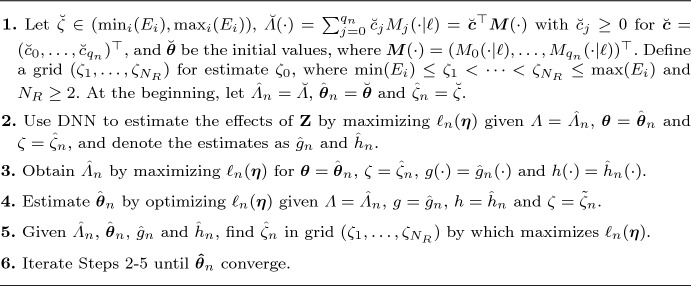
Profiling estimation procedure for parameter ${\boldsymbol{\eta }}$.

Step 1 gives the initial values, and it is recommended to let $\breve{\boldsymbol{\theta }}$ be a zero vector with dimension 2*p* to keep the proportional term from influencing the initial estimation and resulting in overfitting. In Step 2, given the initial values of ${\boldsymbol{\theta }}$ and spline parameters $c_j$’s, the $\hat{W}_j$’s and $\hat{\boldsymbol{\nu }}_j$’s can be obtained by minimizing the loss function using the Pytorch. We estimate $g_0$ and $h_0$ jointly by choosing $p_{K+1}=2$, producing the two-dimensional output $(\hat{g}_n({\textbf{Z}}),\hat{h}_n({\textbf{Z}}))$ for each ${\textbf{Z}}$. Then, in Step 3, fixing the remaining parameters, we estimate $\hat{c}_j$ for $\hat{\Lambda }_n$ and set $\hat{\Lambda }_n(\cdot )=\sum _{i=1}^{q_n}\hat{c}_j M_j(\cdot |\ell )$. In Step 4, we apply the sequential least squares programming to estimate ${\boldsymbol{\theta }}_0$ while keeping the remaining parameters fixed. While a larger $N_R$ in Step 5 enhances the precision of $\zeta _0$, it also raises computational burden. In practice, one should balance accuracy against cost. Finally, in Step 6, if we define $\hat{ \boldsymbol{\theta }}_n^{(j)}$ as the estimation of regression parameters in the *j* iteration and $\Vert \cdot \Vert _\infty$ as the $L^{\infty }$ norm, our iteration procedures stop at the *j*th step when $\Vert \hat{\boldsymbol{\theta }}_n^{(j)}-\hat{\boldsymbol{\theta }}_n^{(j-1)}\Vert _\infty <\epsilon$ for some given $\epsilon >0$.

Some details of the neural network optimization are discussed here. The Adam optimizer estimates the $W_j$ and ${\boldsymbol{\nu }}_j$ of the deep neural network, which is reliable and practical in various models (Kingma and Ba 2014). The Adam optimization algorithm utilizes first-order moment estimates and second-order moment estimates of the gradients to dynamically adjust the learning rates for each parameter. The tuning parameters of the DNN contain the number of hidden layers *K*, the number of neurons $p_k$ in all hidden layers, the number of epochs, the dropout rate and the learning rate. The learning rate is the step size of gradient descent, while the dropout rate refers to the random disregard of partial neurons during training.

In all simulations, based on Model (2), ${\textbf{X}}$ is generated from the Bernoulli distribution with a successful probability of 0.5. The covariate ${\textbf{Z}}$ is generated from a multivariate *t*-distribution $t_5({\boldsymbol{0}},{\boldsymbol{\Sigma }})$ with truncation on [0, 2], where ${\boldsymbol{\Sigma }} = (\sigma _{ij})$ with $\sigma _{ii}=1$ and $\sigma _{ij}=0.5$ for $i\ne j$. The variable *E* is distributed as *N*(2, 1) truncated on the interval [1.5, 2.5]. We set the true values of the regression parameters ${\boldsymbol{\theta }}_0=(\beta _0,\gamma _0)^\top = (-1, 2)^\top$, the change point $\zeta _0 = 2$ and the baseline cumulative hazard function $\Lambda _0(t)=\sqrt{t}/5$. For covariate ${\textbf{Z}}$, we design the following 3 cases with ${\textbf{z}}=(z_1,\dots ,z_5)^\top \in [0,2]^5$:

**Case 1 (Linear): **$$g_0({\textbf{z}})=-z_1/2-z_2/3-z_3/4-z_4/5-z_5/6+0.94$$, $$h_0({\textbf{z}})=-z_1/3-z_2/4-z_3/5-z_4/6-z_5/7+0.71$$,

**Case 2 (Additive): **$$g_0({\textbf{z}})=z_1^2/2+2\log (z_2+1)/5+3\sqrt{z_3}/10+e^{z_4}/5+z_5^3/10-1.62$$, $$h_0({\textbf{z}})=\sin (2\pi z_1)+e^{z_2}/5+3\sqrt{z_3}/5+\log (z_4+1)/3+z_5^2/3-1.38$$,

**Case 3 (Deep): **$$g_0({\textbf{z}})=\sqrt{z_1z_2}/5+z_3^2z_4/4+\log (z_4+1)/3+e^{z_5}/2-1.91$$, $$h_0({{\textbf{z}}})=\{\sqrt{z_1z_2}/5+z_3^2z_4/4+\log (z_4+1)/3+e^{z_5}/2\}^2/5-1.36$$.

All intercept terms are added to satisfy the identifiable conditions ${\mathbb {E}} g_0({\textbf{Z}})=0$ and ${\mathbb {E}} h_0({\textbf{Z}})=0$. In cases 1 and 2, the effects of all covariates on the log cumulative hazard function are linear and additive, respectively, while the effects in Case 3 are more complicated.

To compare with our proposed method (CPDPLCM), we also include an approach that estimates the effect of ${\textbf{Z}}$ using a linear model. This method assumes the form $\Lambda (t|{\textbf{X}}, {\textbf{Z}}, E) = \Lambda _0(t)\exp \{{\boldsymbol{\beta }_0}^\top {\textbf{X}} + \boldsymbol{\varsigma }_0^\top {\textbf{Z}} + ({\boldsymbol{\gamma }}_0^\top {\textbf{X}} + \boldsymbol{\vartheta }_0^\top {\textbf{Z}} + \vartheta _0)I_{\{E >\zeta _0\}}\},$ where $\boldsymbol{\varsigma }_0, \boldsymbol{\vartheta }_0 \in \mathbb {R}^r$ and $\vartheta _0 \in \mathbb {R}$. This approach, referred to as CPCPH, is a direct extension of methods of Huang (1996) and Pons (2003). For both the two methods, we chose $\breve{\zeta }=\frac{1}{n}\sum _{i=1}^n E_i$, $\breve{{\boldsymbol{\theta }}}=(\breve{\beta },\breve{\gamma })^{\top }=(0,0)^\top$ and $\breve{\boldsymbol{c}}=(\breve{c}_0,\dots ,\breve{c}_{q_n})^\top$ with $\breve{c}_j=0.1$ for $j=0,\dots ,q_n$ to be the initial values. The sample size for each case was $n = 1000, 2000$, or 4000, respectively, and 200 replications were performed.

We first describe the evaluation criteria for the estimates. The estimates of regression parameters are evaluated by the estimated biases (Bias), sampling standard errors (SSE), sampling means of the estimated standard error estimates (ESE), and the 95% empirical coverage probabilities (CP). To evaluate the performance of DNN functions, we consider the relative error (RE), defined as

$$\begin{aligned} RE(\hat{g})=\left\{ \frac{\frac{1}{n_t}\sum _{i=1}^{n_t}\{ \{\hat{g}({\textbf{Z}}_i)-\frac{1}{n_t}\sum _{i=1}^{n_t}\hat{g}({\textbf{Z}}_i)\} - g_0({\textbf{Z}}_i)\}^2}{\frac{1}{n_t}\sum _{i=1}^{n_t}\{g_0({\textbf{Z}}_i)\}^2} \right\} ^{1/2} \end{aligned}$$

with its SSE, where $\hat{g}$ and $g_0$ are evaluated at the test set’s covariates $\{\textbf{Z}_i,i=1,\dots ,n_t\}$, with $n_t=200$. We also consider the prediction error for the *i*th test observation, defined as $\text {Error}_i(\hat{g})=\hat{g}({\textbf{Z}_i})-g_0({\textbf{Z}_i})$. Obtain the change point estimate $\hat{\zeta }_n$ by searching a grid with a given gap; in this study, we choose the gap of 0.01 to balance the accuracy and the computational cost. The simulation results include the estimated bias, the length of the 95% empirical confidence interval (95% CI Length), and the SSE of the estimation. Specifically, for *M* replications, denote $\boldsymbol{\zeta }_M=(\hat{\zeta }^{(1)}_n,\dots ,\hat{\zeta }^{(M)}_n)$ be the estimation collection of $\zeta _0$, the 95% CI Length is defined as the difference of the upper 2.5% quantile and the lower 2.5% quantile of $\boldsymbol{\zeta }_M$.

**Table 1:** Estimation of the regression parameters ${\boldsymbol{\theta }}_0=(\beta _0,\gamma _0)^\top$

Model	*n*	Para	CPDPLCM				CPCPH			
			Bias	SSE	ESE	CP	Bias	SSE	ESE	CP
Case 1	1000	$\beta$	$-$ 0.018	0.193	0.197	0.965	$-$ 0.020	0.197	0.189	0.945
		$\gamma$	0.088	0.256	0.268	0.935	0.061	0.251	0.260	0.955
	2000	$\beta$	$-$ 0.003	0.139	0.127	0.935	$-$ 0.006	0.139	0.132	0.935
		$\gamma$	0.061	0.189	0.182	0.940	0.026	0.186	0.179	0.950
	4000	$\beta$	0.002	0.094	0.096	0.960	$-$ 0.001	0.093	0.093	0.945
		$\gamma$	0.029	0.132	0.126	0.950	0.013	0.129	0.125	0.970
Case 2	1000	$\beta$	0.003	0.237	0.247	0.950	0.011	0.216	0.221	0.965
		$\gamma$	0.059	0.329	0.324	0.940	$-$ 0.109	0.315	0.316	0.920
	2000	$\beta$	0.024	0.160	0.155	0.935	0.029	0.156	0.151	0.935
		$\gamma$	$-$ 0.041	0.224	0.219	0.930	$-$ 0.135	0.212	0.213	0.875
	4000	$\beta$	$-$ 0.012	0.104	0.115	0.950	0.054	0.095	0.104	0.945
		$\gamma$	$-$ 0.008	0.153	0.154	0.945	$-$ 0.189	0.140	0.146	0.745
Case 3	1000	$\beta$	$-$ 0.035	0.255	0.252	0.930	0.061	0.238	0.239	0.950
		$\gamma$	0.061	0.346	0.367	0.960	$-$ 0.325	0.339	0.368	0.845
	2000	$\beta$	$-$ 0.033	0.181	0.187	0.950	0.095	0.152	0.162	0.940
		$\gamma$	0.043	0.273	0.290	0.955	$-$ 0.400	0.215	0.246	0.610
	4000	$\beta$	$-$ 0.022	0.117	0.113	0.940	0.121	0.101	0.111	0.845
		$\gamma$	0.003	0.182	0.176	0.955	$-$ 0.467	0.161	0.169	0.195

As reported in Table 1, the estimators $\hat{{\beta }}_n$ and $\hat{{\gamma }}_n$ exhibit improved performance as the sample size increases in all cases. For the proposed CPDPLCM method, the results indicate that $\hat{{\beta }}_n$ and $\hat{{\gamma }}_n$ are unbiased and asymptotically normal, in accordance with Theorem 5. Moreover, the procedure in Theorem 4 for estimating the asymptotic variance is validated as reliable. The 95% coverage probabilities for both $\hat{{\beta }}_n$ and $\hat{{\gamma }}_n$ under the proposed method are generally close to 0.95 as the sample size increases. Note that $\hat{{\beta }}_n$ is estimated more accurately than $\hat{{\gamma }}_n$ because the change-point effect does not occur for every observation, reducing the information available for estimating $\hat{{\gamma }}_n$. In addition, in Cases 2 and 3, the CPCPH method exhibits substantial bias due to model misspecification. Although Case 1 is much simpler and satisfies the assumptions of CPCPH, our method remains highly competitive, with only a slight loss of efficiency. Overall, the proposed approach is flexible for estimating regression parameters and outperforms CPCPH.

**Table 2:** Evaluation of relative errors for $\hat{g}$ and $\hat{h}$ on the test data

Scenario	*n*	$\hat{g}$				$\hat{h}$			
		CPDPLCM		CPCPH		CPDPLCM		CPCPH	
		RE	SSE	RE	SSE	RE	SSE	RE	SSE
Case 1	1000	0.240	0.044	0.295	0.086	0.298	0.106	0.484	0.150
	2000	0.227	0.037	0.222	0.049	0.272	0.086	0.348	0.101
	4000	0.186	0.026	0.181	0.033	0.218	0.056	0.275	0.073
Case 2	1000	0.270	0.038	0.246	0.038	0.556	0.053	0.561	0.082
	2000	0.229	0.024	0.217	0.017	0.487	0.056	0.482	0.078
	4000	0.194	0.019	0.205	0.009	0.414	0.051	0.474	0.062
Case 3	1000	0.221	0.036	0.303	0.025	0.603	0.068	0.774	0.092
	2000	0.185	0.018	0.288	0.014	0.585	0.064	0.762	0.068
	4000	0.162	0.013	0.283	0.008	0.542	0.055	0.756	0.046

**Fig. 1:**
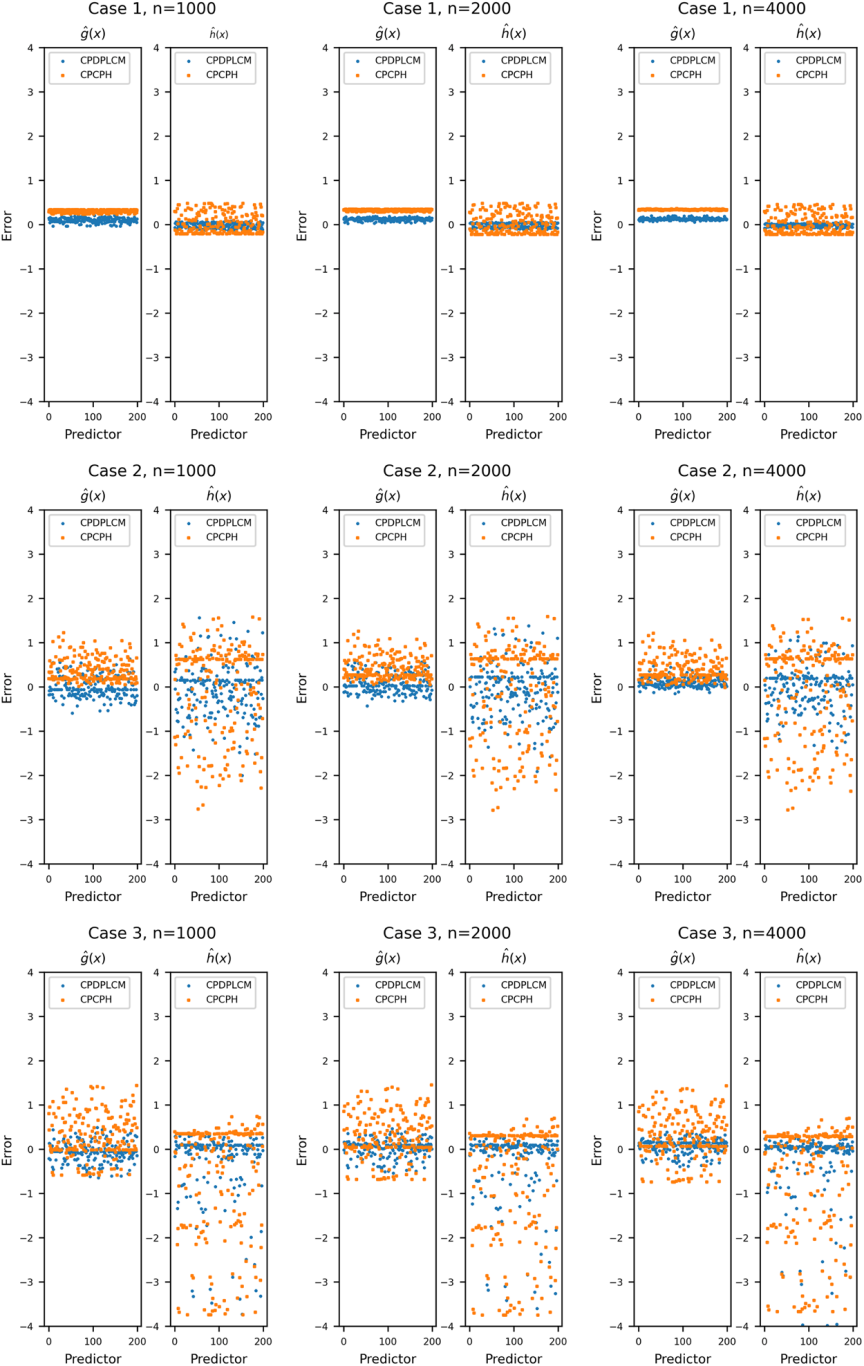
Prediction errors of the DNN function *g* and *h* estimated by the proposed CPDPLCM (circle) and CPCPH (square) on test data (sample size = 200) with sample sizes $n=1000,2000$ and 4000 for three different cases

Table 2 summarizes the relative errors (REs) and their standard deviations on the test set, and Fig. 1 visualizes the corresponding prediction errors. Both the RE and its SSE decrease as the sample size increases. The relative error of $\hat{h}$ exceeds that of $\hat{g}$, mirroring the behavior observed for $\hat{\boldsymbol{\beta }}$ versus $\hat{\boldsymbol{\gamma }}$. When the true functions correspond to Case 1, the proposed method is slightly less efficient than the perfectly specified CPCPH in estimating g0 and h0. In contrast, for Cases 2 and 3, as sample size grows, our method outperforms CPCPH, yielding smaller REs and prediction errors and exhibiting greater flexibility.

Table 3 presents the simulation results for the change-point estimates. When the identifiability conditions are satisfied, both methods produce valid estimates, corroborating the asymptotic independence established in Theorem 3.3. For both methods, performance improves with increasing sample size. The bias of the change-point estimates is smaller than that of the regression parameters, reflecting the faster convergence rate predicted by Theorem 3.5. Across settings, the accuracy of the change-point estimates degrades slightly as the covariate effects become more complex.

Overall, the CPDPLCM method performs well across all scenarios considered and demonstrates strong flexibility to varying model structures.

**Table 3:** Evaluation of the estimation of the change point $\zeta _0$

Scenario	*n*	CPDPLCM			CPCPH		
		Bias	95% CI length	SSE	Bias	95% CI length	SSE
Case 1	1000	$-$ 0.0003	0.0800	0.0114	$-$ 0.0001	0.0800	0.0110
	2000	$-$ 0.0005	0.0400	0.0050	$-$ 0.0002	0.0400	0.0047
	4000	$-$ 0.0003	0.0200	0.0018	$-$ 0.0005	0.0200	0.0017
Case 2	1000	0.0043	0.2000	0.0270	0.0001	0.1600	0.0205
	2000	0.0028	0.1000	0.0142	0.0020	0.0700	0.0114
	4000	$-$ 0.0007	0.0500	0.0058	$-$ 0.0007	0.0500	0.0058
Case 3	1000	0.0213	0.3400	0.0545	0.0018	0.2900	0.0471
	2000	0.0114	0.1900	0.0262	0.0009	0.1500	0.0208
	4000	0.0058	0.1500	0.0190	$-$ 0.0029	0.1000	0.0149

## Applications to Rotterdam breast cancer data

### Data and model

We applied our proposed methods to a breast cancer dataset comprising records for 2,982 primary breast cancer patients from the Rotterdam Tumor Bank, as used by Royston and Altman (2013). They also recommended using the German Breast Cancer Study Group (GBSG) data, which contain 686 complete patient records, as the test set. Both datasets are included in the R package survival and are publicly available at https://cran.r-project.org/web/packages/survival/index.html.

In our study, we use the Rotterdam data to train the model and aim to detect the change-point effect of chemotherapy across ages in preventing breast cancer relapse. Prior work (Keogh and Morris 2018; Chen et al., 2023) suggests that the Cox model is appropriate, and the censoring mechanism is typically treated as right censoring. However, event times may be imprecise and subject to bias (e.g., delayed detection), so treating them as interval-censored is more general and often more robust. A summary of the variables, with minor preprocessing for computational convenience, is provided in the following table.

**Table 4:** A summary of the Rotterdam data

Variable	Description	Min	Mean	Max
Rtime	Time to relapse or last follow-up (year)	0.0986	5.7477	19.2959
Recur	0 = no relapse, 1 = relapse	0.0000	0.5091	1.0000
Chemo	0 = no chemotherapy, 1 = experienced	0.0000	0.1945	1.0000
Age	Age at surgery (year)	24.0000	55.0600	90.0000
Meno	0 = premenopausal, 1 = postmenopausal	0.0000	0.5600	1.0000
Size	Tumor size (0 = “$\le$20”, 1 =“20–50”, 2 =“$\ge$50”)	0.0000	0.6368	2.0000
Grade	Differentiation grade (take values 2 and 3)	2.0000	2.7340	3.0000
Nodes	Number of positive lymph nodes	0.0000	2.7120	34.0000
Pgr	Progesterone receptors ($10^2$ fmol/L)	0.0000	1.6180	50.0400
Er	Estrogen receptors ($10^2$ fmol/L)	0.0000	1.6660	32.7500
Hormon	Hormonal treatment (0 = no, 1 = yes)	0.0000	0.1137	1.0000

In this study, we use the binary variable chemo as *X*, and define the event of interest as breast cancer relapse, with observation time *U* and censoring indicator $\Delta$ taken from rtime and recur, respectively. As noted by Lee et al. (2020), in cancer research the primary outcomes are overall survival or disease-free survival, for which age is often a key prognostic factor; accordingly, we take the change-point variable E to be age. The 7-dimensional covariate Z comprises the remaining variables. According to Table 4, patients who received chemotherapy are a minority (about 20%). Patient ages range from 24 to 90, with middle-aged and older patients predominating. Approximately half of the patients experience relapse, yielding a censoring rate of about 50%.

The model relating relapse time to chemotherapy is specified as

$$\begin{aligned} \Lambda (t|X,{\textbf{Z}},E)=\Lambda _0(t)\exp \left\{ \beta _0 X+g_0({\textbf{Z}})+\{\gamma _0 X+h_0({\textbf{Z}})\}I_{\{E>\zeta _0\}}\right\} . \end{aligned}$$

In this model, $\beta _0$ is the baseline effect of chemotherapy on relapse prevention, while $\gamma _0$ captures the additional effect that manifests beyond the age threshold $\zeta _0$. The covariate vector ${\textbf{Z}}$ represents other control variables, whose effect is modeled by $g_0(\cdot )$; analogous to the treatment effect, the impact of ${\textbf{Z}}$ may vary with age, and this differential is captured by $h_0(\cdot )$. For comparison, we also apply the CPCPH method.

### Results

This section presents the empirical results based on the real data described above. Before conducting the regression analysis, we first implement the testing procedure in Sect. 4. For computational convenience, we randomly permute the change-point covariate *E* 1,000 times, set $k=5$ in the statistic $\textrm{SUP}_k$ , and obtain a *p*-value of 0.007, indicating the presence of a change point and yielding an estimate of $\hat{\zeta }_n=48$.

As shown in Table 5, the *p*-values obtained by our method are below 0.05, whereas those from the CPCPH method exceed 0.05, indicating that our estimates are statistically significant while CPCPH’s are not. Moreover, the 95% confidence interval produced by our method excludes 0, whereas that of CPCPH includes 0, underscoring the greater reliability of our interval estimates. Furthermore, the hazard ratio of patients older than 48 versus those younger than 48 is $\exp (0.3508)\approx 142.02\%$ on average.

**Table 5:** Estimation results for regression parameter $(\beta ,\gamma )$

Methods	Para	Value	SE	*p* value	95% CI
CPDPLCM	$\beta$	$-$ 0.5832	0.1247	$<10^{-5}$	[$-$ 0.8275, $-$ 0.3388]
	$\gamma$	0.3508	0.1508	0.0100	[0.0552, 0.6464]
CPCPH	$\beta$	$-$ 0.0068	0.1279	0.4788	[$-$ 0.2575, 0.2439]
	$\gamma$	$-$ 0.0201	0.1504	0.4469	[$-$ 0.3150, 0.2748]

The reduced immunological recovery and the menopause are two potential explanations for the change-point effect. First, it is well-known that chemotherapy is harmful both to cancer cells and to normal cells, so it is more difficult for elderly patients to recover from the damage caused by chemotherapy. In addition, according to Ossewaarde et al. (2005), menopause is strongly associated with an increased risk of breast cancer. They discovered that the average age at menopause is $49.0 \pm 4.5$ years, suggesting that the effect of the change point may come from menopause.

In conclusion, our method effectively detects a shift in chemotherapy effects before and after age 48 and yields reliable 95% confidence interval estimates. We find that chemotherapy substantially reduces recurrence risk across all age groups; however, its preventive effectiveness diminishes among patients older than 48, indicating a change-point effect in efficacy.

## Concluding remarks

This study focuses on the Cox model with a change point at the covariate for the current status data, proposes a DNN-based estimation method, gives the semiparametric validity of the maximum likelihood estimation, and provides a computational scheme. This method is an effective tool for analyzing interval-censored survival data and identifying significant changes in risk factors, especially when the effects of covariates or nuisance parameters are complex.

This method can be directly extended to a class of generalized partial linear models, such as the transformation model (Kosorok and Song 2007) and the Cox model with right censoring (Pons 2003). Furthermore, the assumption that all covariates are affected by the change-point effect of a single univariate variable is restrictive. Thus, a multivariate $\boldsymbol{E}=(E_1,\dots ,E_q)^\top$ with threshold $\boldsymbol{\zeta }=(\zeta _1,\dots ,\zeta _q)^\top$ could be considered, and variable selection techniques may help detect these more complex change-point effects. In addition, heterogeneity across observations, common in precision medicine, suggests that the change point may be individualized; it may therefore be valuable to consider models with individualized change-point effects and to design personalized therapies.

Another challenging issue is how to effectively verify model identifiability. Identifiability is crucial for ensuring estimator consistency; however, current approaches (e.g., Kosorok and Song (2007)) rely heavily on random shuffling and sampling, which may be ineffective when training a DNN and consume substantial computing resources. An optimal approach is to avoid resampling and repeated DNN training to save time. We will explore advances on this issue in future work.

## Supplementary Information

Below is the link to the electronic supplementary material.

Supplementary file 1 (pdf 815 KB)
