# Supplementary material for: Deep learning for the change-point Cox model with current status data
Source: Lifetime Data Anal. 2026 Feb 9;32(1):14. doi: 10.1007/s10985-026-09689-y (PMC12886198; doi:10.1007/s10985-026-09689-y)
Supplement: Supplementary file 1 — (pdf 815 KB) [file 10985_2026_9689_MOESM1_ESM.pdf]

The Supplementary Information for "Deep learning for the change-point Cox model with current status data"

1 Appendix A

In the appendices, we provide some useful lemmas and detailed proofs of some theoretical results in the paper. For any q-dimensional vector $\mathbf{a} = (a_1 \ldots, a_q)^\top \in \mathbb{R}^q$, let $\|\mathbf{a}\| = \sqrt{\sum_{i=1}^q a_i^2}$. We use D to denote a generic positive finite constant that may differ in different uses and define $\mathbb{R}_D^p = \{\boldsymbol{\beta} \in \mathbb{R}^p : \|\boldsymbol{\beta}\| < D\}$ for every finite fixed p, $\mathcal{F}_D = \{\sum_{j=1}^{q_n} c_j M_j(t|\ell) : \ell \in \mathbb{N}, 0 \le c_j \le D$, for $j = 1, \ldots, q_n, t \in [C_u, D_u]\}$ and $\mathcal{G}_D = \mathcal{G}\{s, K, \boldsymbol{p}, D\}$. For any q-dimensional vector $\mathbf{a} = (a_1 \ldots, a_q)^\top \in \mathbb{R}^q$, let $\|\mathbf{a}\| = \sqrt{\sum_{i=1}^q a_i^2}$. Also, $\xrightarrow{P}$ denotes converge in probability, $\xrightarrow{d}$ represents converge in distribution, and $\mathbf{X} \overset{d}{=} \mathbf{Y}$ means the random variables $\mathbf{X}$ and $\mathbf{Y}$ share the same distribution. Denotes $a_n \lesssim b_n$ as $a_n \le cb_n$ and $a_n \gtrsim b_n$ as $a_n \ge cb_n$ for some constant $c > 0$ respectively, and $a_n \asymp b_n$ indicates $a_n \lesssim b_n$ and $a_n \gtrsim b_n$.

1.1 Technical Lemmas

Lemma 1. *Under* (C1)-(C7)*, denote* $\boldsymbol{\eta}_{0,n} = (\Lambda_{0,n}, \boldsymbol{\beta}_0, g_{0,n}, \boldsymbol{\gamma}_0, h_{0,n}, \zeta_0)$*. Let*

$$\mathcal{B}_\delta = \left\{\boldsymbol{\eta} \in \mathcal{F}_D \times \mathbb{R}_D^p \times \mathcal{G}_D \times \mathbb{R}_D^p \times \mathcal{G}_D \times \mathbb{R}_D : d(\boldsymbol{\eta}, \boldsymbol{\eta}_{0,n}) \le \delta\right\}$$

for some $D > 0$*. Let* $\mathbb{G}_n = \sqrt{n}(\mathbb{P}_n - \mathbb{P})$*, then*

$$\mathbb{E}^* \sup_{\boldsymbol{\eta} \in \mathcal{B}_\delta} |\mathbb{G}_n\{m_{\boldsymbol{\eta}}(\boldsymbol{V}) - m_{\boldsymbol{\eta}_{0,n}}(\boldsymbol{V})\}| = O\left(\delta\sqrt{s\log\frac{L}{\delta}} + \frac{s}{\sqrt{n}}\log\frac{L}{\delta}\right),$$

where $\mathbb{E}^*$ *denotes the outer expectation and* $L = K\prod_{k=0}^K (p_k + 1)\sum_{k=0}^K p_k p_{k+1}$*.*

Lemma 2. *Under* (C3)-(C7)*, we have*

$$\ell(\boldsymbol{\eta}) - \ell(\boldsymbol{\eta}_0) \asymp -d^2(\boldsymbol{\eta}, \boldsymbol{\eta}_0)$$

for all $\boldsymbol{\eta} \in \{\boldsymbol{\eta} : d(\boldsymbol{\eta}, \boldsymbol{\eta}_0) < \epsilon\}$ *with some small* $\epsilon > 0$*.*

Lemma 3. *Under* (C1)-(C10)*, if the estimate of* $(g, h, \Lambda) \in \mathcal{G} \times \mathcal{G} \times \mathcal{F}$ *satisfies*

$$\mathbb{P}\left(\sup_{(\zeta,\boldsymbol{\theta}) \in \mathbb{R}_D^{2p+1}} \ell_n(\zeta, \boldsymbol{\theta}|g, h, \Lambda) - \sup_{(\zeta,\boldsymbol{\theta}) \in \mathbb{R}_D^{2p+1}} \ell_n(\zeta, \boldsymbol{\theta}|g_0, h_0, \Lambda_0)\right) \le u$$

for any $0 < u < 1$ *with sufficiently large* n *and given* $D > 0$*, there exists*

$$\lim_{A\to\infty}\limsup_{n\to\infty} \mathbb{P}(n|\hat{\zeta}_n - \zeta_0| > A) = 0\,, \quad \lim_{A\to\infty}\limsup_{n\to\infty} \mathbb{P}(n^{1/2}\|\hat{\boldsymbol{\theta}}_n - \boldsymbol{\theta}_0\| > A) = 0\,.$$

1.2 Proofs of Lemmas and Theorems

Proof of Theorem 1

Consider that there are two sets of parameters $\boldsymbol{\eta}_1$ and $\boldsymbol{\eta}_0$ such that $m_{\boldsymbol{\eta}_1}(\boldsymbol{V}) = m_{\boldsymbol{\eta}_0}(\boldsymbol{V})$ a.s. for every $\boldsymbol{V}$. Then for all $(\Delta, \boldsymbol{V})$,

$$\begin{aligned} 0 &= m_{\boldsymbol{\eta}_1}(\boldsymbol{V}) - m_{\boldsymbol{\eta}_0}(\boldsymbol{V}) \\ &= \Delta\log\frac{1 - \exp\{-\boldsymbol{h}_{\boldsymbol{\eta}_1}(\boldsymbol{V})\}}{1 - \exp\{-\boldsymbol{h}_{\boldsymbol{\eta}_0}(\boldsymbol{V})\}} - (1-\Delta)(\boldsymbol{h}_{\boldsymbol{\eta}_1}(\boldsymbol{V}) - \boldsymbol{h}_{\boldsymbol{\eta}_0}(\boldsymbol{V}))\,. \end{aligned}$$

Take $\Delta = 0$, $\boldsymbol{h}_{\boldsymbol{\eta}_1}(\boldsymbol{V}) = \boldsymbol{h}_{\boldsymbol{\eta}_0}(\boldsymbol{V})$ a.s., thus for all $\boldsymbol{V} = (U, \mathbf{X}, \mathbf{Z}, E)$, almost surely there exists

$$\log \frac{\Lambda_1(U)}{\Lambda_0(U)} + (\boldsymbol{\beta}_1 - \boldsymbol{\beta}_0)^\top \mathbf{X} + g_1(\mathbf{Z}) - g_0(\mathbf{Z}) + \{\boldsymbol{\gamma}_1^\top \mathbf{X} + h_1(\mathbf{Z})\} I_{\{E > \zeta_1\}} - \{\boldsymbol{\gamma}_0^\top \mathbf{X} + h_0(\mathbf{Z})\} I_{\{E > \zeta_0\}} = 0 \,.$$

We first demonstrate $\zeta_1 = \zeta_0$. Otherwise, without loss of generality, assume $\zeta_1 < \zeta_0$, and choose $E > \zeta_0$,

$$\log \frac{\Lambda_1(U)}{\Lambda_0(U)} + (\boldsymbol{\beta}_1 - \boldsymbol{\beta}_0 + \boldsymbol{\gamma}_1 - \boldsymbol{\gamma}_0)^\top \mathbf{X} + g_1(\mathbf{Z}) - g_0(\mathbf{Z}) + h_1(\mathbf{Z}) - h_0(\mathbf{Z}) = 0 \ \ a.s. \ . \tag{1}$$

Then choosing $\zeta_1 < E < \zeta_0$, there exists

$$\log \frac{\Lambda_1(U)}{\Lambda_0(U)} + (\boldsymbol{\beta}_1 + \boldsymbol{\gamma}_1 - \boldsymbol{\beta}_0)^\top \mathbf{X} + g_1(\mathbf{Z}) + h_1(\mathbf{Z}) - g_0(\mathbf{Z}) = 0 \ \ a.s.. \tag{2}$$

Combining (1) and (2), we have $\boldsymbol{\gamma}_0^\top \mathbf{X} + h_0(\mathbf{Z}) = 0$ a.s., which contradicts the condition (C9). $\zeta_1 > \zeta_0$ shows the same contradiction. Hence $\zeta_1 = \zeta_0$.

The identifiability of the rest of the parameters is shown as follows. Choose $E < \zeta_0$, for all $(\mathbf{X}, \mathbf{Z}) \in \mathbb{R}^p \times [0,1]^r$ and $U \in [0, \tau]$, there exists

$$\log \frac{\Lambda_1(U)}{\Lambda_0(U)} + (\boldsymbol{\beta}_1 - \boldsymbol{\beta}_0)^\top \mathbf{X} + g_1(\mathbf{Z}) - g_0(\mathbf{Z}) = 0 \ \ a.s. \ .$$

Let $\mathbf{X} = 0$, we have $\log \Lambda_1(U) + g_1(\mathbf{Z}) = \log \Lambda_0(U) + g_0(\mathbf{Z})$ almost surely. Then we take the expectation of $\mathbf{Z}$, by (C8) and (C9), we have $\Lambda_1(\cdot) = \Lambda_0(\cdot)$, hence $g_1(\mathbf{Z}) = g_0(\mathbf{Z})$ almost surely for all $\mathbf{Z} \in [0,1]^r$, indicating that $g_1(\cdot) = g_0(\cdot)$ and $\boldsymbol{\beta}_1 = \boldsymbol{\beta}_0$. Choose $E > \zeta_0$, we have

$$(\boldsymbol{\gamma}_1 - \boldsymbol{\gamma}_0)^\top \mathbf{X} + (h_1(\mathbf{Z}) - h_0(\mathbf{Z})) = 0 \ \ a.s. \ ,$$

which similarly yields $\boldsymbol{\gamma}_1 = \boldsymbol{\gamma}_0$ and $h_1(\cdot) = h_0(\cdot)$ by conditions (C8) and (C9). Hence all parameters are identifiable. □

Proof of Lemma 1

Denote $\mathcal{D}_\delta = \{m_{\boldsymbol{\eta}}(\boldsymbol{V}) - m_{\boldsymbol{\eta}_{0,n}}(\boldsymbol{V}) : \boldsymbol{\eta} \in \mathcal{B}_\delta\}$ and define $\|\mathbb{G}\|_{\mathcal{G}_\delta} = \sup_{f \in \mathcal{D}_\delta} |\mathbb{G}_n f|$. Note that for any $\boldsymbol{\eta}_1, \boldsymbol{\eta}_2 \in \mathcal{B}_\delta$,

$$\begin{aligned}
\mathbb{E}[m_{\boldsymbol{\eta}_1}(\boldsymbol{V}) - m_{\boldsymbol{\eta}_2}(\boldsymbol{V})]^2 &\lesssim \mathbb{E}[\boldsymbol{h}_{\boldsymbol{\eta}_1}(\boldsymbol{V}) - \boldsymbol{h}_{\boldsymbol{\eta}_2}(\boldsymbol{V})]^2 \\
&\lesssim \mathbb{E}\left\{\|\Lambda_1(U) - \Lambda_2(U)\|^2 + \|\boldsymbol{\beta}_1 - \boldsymbol{\beta}_2\|^2 + \|g_1(\mathbf{Z}) - g_2(\mathbf{Z})\|^2 \right. \\
&\left. + \|\boldsymbol{\gamma}_1 - \boldsymbol{\gamma}_2\|^2 + \|h_1(\mathbf{Z}) - h_2(\mathbf{Z})\|^2 + |\zeta_1 - \zeta_2|\right\} \\
&= d^2(\boldsymbol{\eta}_1, \boldsymbol{\eta}_2) \,.
\end{aligned}$$

According to Lu and Song (2015), the logarithm of the bracketing number of $\{\Lambda \in \mathcal{F}_D : \|\Lambda - \Lambda_{0,n}\|_{L^2} \le \delta\}$ is controlled by

$$\log \mathcal{N}_{[]}(\epsilon, \{\Lambda \in \mathcal{F}_D : \|\Lambda - \Lambda_{0,n}\|_{L^2} \le \delta\}, L^2(P)) \lesssim c q_n \log \frac{\delta}{\epsilon} \,.$$

By Zhong et al. (2022), for $g(\cdot)$ and $h(\cdot)$,

$$\log \mathcal{N}_{[]}(\epsilon, \mathcal{G}_\delta, L^2(P)) \lesssim s \log \frac{L}{\epsilon} \,.$$

It is known that the space $\{\boldsymbol{\beta} : \|\boldsymbol{\beta} - \boldsymbol{\beta}_0\| \le \delta\}$ can be covered by $c(\delta/\epsilon)^p$ balls with radius ϵ where $c > 0$ is some constant. Thus, the log-bracketing entropy of p-dimensional $\boldsymbol{\beta}$ and $\boldsymbol{\gamma}$ is bounded by

$$\log \mathcal{N}_{[]}(\epsilon, \{\boldsymbol{\beta} \in \mathbb{R}_D^p : \|\boldsymbol{\beta} - \boldsymbol{\beta}_0\| < \delta\}, L^2(P)) \lesssim p \log \frac{\delta}{\epsilon} \,.$$

The entropy of the change-point parameter $E \in \mathbb{R}_D$ satisfies

$$\log \mathcal{N}_{[]}(\epsilon, \{\zeta \in \mathbb{R}_D : |\zeta - \zeta_0| < \delta\}, L^2(P)) \lesssim \log \frac{\delta}{\epsilon} \,.$$

Then for $p, q_n \le s$ and $\delta \le L$,

$$\log \mathcal{N}_{[]}(\epsilon, \mathcal{D}_\delta, L^2(\mathbb{P})) \lesssim 2p \log \frac{\delta}{\epsilon} + q_n \log \frac{\delta}{\epsilon} + 2s \log \frac{L}{\epsilon} + \log \frac{\delta}{\epsilon} \lesssim s \log \frac{\delta}{\epsilon} \,,$$

and the bracketing integral $\mathcal{D}_\delta$ is obtained by

$$\begin{aligned}J_{[]}(\epsilon,\mathcal{D}_\delta,L^2(\mathbb{P})) &:= \int_0^\delta \sqrt{1+\log \mathcal{N}_{[]}(\epsilon,\mathcal{D}_\delta,L^2(\mathbb{P}))}d\epsilon \\ &\lesssim \int_0^\delta \sqrt{s\log\frac{L}{\epsilon}}d\epsilon \\ &\asymp \int_0^\delta \sqrt{s\log\frac{L}{\delta}}d\epsilon \\ &= \delta\sqrt{s\log\frac{L}{\delta}}\,.\end{aligned}$$

Thus Lemma 3.4.3 in van der Vaart and Wellner (1996) yields

$$\begin{aligned}\mathbb{E}^*\|\mathbb{G}_n\|_{\mathcal{D}_\delta} &\lesssim J_{[]}(\epsilon,\mathcal{D}_\delta,L^2(\mathbb{P}))\left\{1+\frac{J_{[]}(\epsilon,\mathcal{D}_\delta,L^2(\mathbb{P}))}{\delta^2\sqrt{n}}\right\} \\ &\lesssim \delta\sqrt{s\log\frac{L}{\delta}}+\frac{s}{\sqrt{n}}\log\frac{L}{\delta}\,.\end{aligned}$$

□

Proof of Lemma 2

Let $\delta_1(\boldsymbol{V}) = 1-\exp(-\boldsymbol{h}_{\boldsymbol{\eta}_1}(\boldsymbol{V}))$ and $\delta_0(\boldsymbol{V}) = 1-\exp(-\boldsymbol{h}_{\boldsymbol{\eta}_0}(\boldsymbol{V}))$, and in $\{\boldsymbol{\eta} : d(\boldsymbol{\eta},\boldsymbol{\eta}_0) < \varepsilon\}$,

$$\begin{aligned}\ell(\boldsymbol{\eta})-\ell(\boldsymbol{\eta}_0) =&\mathbb{E}\left\{\Delta\log\frac{1-\exp(-\boldsymbol{h}_{\boldsymbol{\eta}}(\boldsymbol{V}))}{1-\exp(-\boldsymbol{h}_{\boldsymbol{\eta}_0}(\boldsymbol{V}))}+(1-\Delta)\log\frac{\exp(-\boldsymbol{h}_{\boldsymbol{\eta}}(\boldsymbol{V}))}{\exp(-\boldsymbol{h}_{\boldsymbol{\eta}_0}(\boldsymbol{V}))}\right\} \\ =&\mathbb{E}\left\{\Delta\log\left[1+\frac{\exp(-\boldsymbol{h}_{\boldsymbol{\eta}_0}(\boldsymbol{V}))-\exp(-\boldsymbol{h}_{\boldsymbol{\eta}}(\boldsymbol{V}))}{1-\exp(-\boldsymbol{h}_{\boldsymbol{\eta}_0}(\boldsymbol{V}))}\right]-(1-\Delta)\log\frac{\exp(-\boldsymbol{h}_{\boldsymbol{\eta}}(\boldsymbol{V}))}{\exp(-\boldsymbol{h}_{\boldsymbol{\eta}_0}(\boldsymbol{V}))}\right\} \\ =&\mathbb{E}\left\{(1-\exp(-\boldsymbol{h}_{\boldsymbol{\eta}_0}(\boldsymbol{V})))\frac{\exp(-\boldsymbol{h}_{\boldsymbol{\eta}_0}(\boldsymbol{V}))-\exp(-\boldsymbol{h}_{\boldsymbol{\eta}}(\boldsymbol{V}))}{1-\exp(-\boldsymbol{h}_{\boldsymbol{\eta}_0}(\boldsymbol{V}))}\right. \\ &\left.-\exp(-\boldsymbol{h}_{\boldsymbol{\eta}_0}(\boldsymbol{V}))\log\frac{\exp(-\boldsymbol{h}_{\boldsymbol{\eta}}(\boldsymbol{V}))}{\exp(-\boldsymbol{h}_{\boldsymbol{\eta}_0}(\boldsymbol{V}))}\right\} \\ =&-\mathbb{E}\left\{\exp(-\boldsymbol{h}_{\boldsymbol{\eta}}(\boldsymbol{V}))-\exp(-\boldsymbol{h}_{\boldsymbol{\eta}_0}(\boldsymbol{V}))-[-\exp(-\boldsymbol{h}_{\boldsymbol{\eta}_0}(\boldsymbol{V}))][\boldsymbol{h}_{\boldsymbol{\eta}}(\boldsymbol{V})-\boldsymbol{h}_{\boldsymbol{\eta}_0}(\boldsymbol{V})]\right\} \\ \asymp&-\mathbb{E}[\boldsymbol{h}_{\boldsymbol{\eta}}(\boldsymbol{V})-\boldsymbol{h}_{\boldsymbol{\eta}_0}(\boldsymbol{V})]^2\,.\end{aligned}$$

According to the fundamental inequality, without loss of generality, assuming $\zeta > \zeta_0$,

$$\begin{aligned}\mathbb{E}[\boldsymbol{h}_{\boldsymbol{\eta}}(\boldsymbol{V})-\boldsymbol{h}_{\boldsymbol{\eta}_0}(\boldsymbol{V})]^2 &\lesssim \mathbb{E}\{(\log\Lambda(U)-\log\Lambda_0(U))+(\boldsymbol{\beta}-\boldsymbol{\beta}_0)^\top\mathbf{X}+(g(\mathbf{Z})-g_0(\mathbf{Z})) \\ &+(\boldsymbol{\gamma}-\boldsymbol{\gamma}_0)^\top\mathbf{X}I_{\{E>\zeta_0\}}+(h(\mathbf{Z})-h_0(\mathbf{Z}))I_{\{E>\zeta_0\}}-(\boldsymbol{\gamma}^\top\mathbf{X}+h(\mathbf{Z}))I_{\{\zeta_0<E\leq\zeta\}}\}^2 \\ &\lesssim \mathbb{E}\{(\log\Lambda(U)-\log\Lambda_0(U))^2+(\boldsymbol{\beta}-\boldsymbol{\beta}_0)^\top\mathbf{X}\mathbf{X}^\top(\boldsymbol{\beta}-\boldsymbol{\beta}_0)+(g(\mathbf{Z})-g_0(\mathbf{Z}))^2 \\ &+(\boldsymbol{\gamma}-\boldsymbol{\gamma}_0)^\top\mathbf{X}\mathbf{X}^\top(\boldsymbol{\gamma}-\boldsymbol{\gamma}_0)I_{\{E>\zeta_0\}}+(h(\mathbf{Z})-h_0(\mathbf{Z}))^2I_{\{E>\zeta_0\}} \\ &+(\boldsymbol{\gamma}^\top\mathbf{X}+h(\mathbf{Z}))^2(F_E(\zeta)-F_E(\zeta_0))\} \\ &\lesssim \mathbb{E}\{(\|\Lambda(U)-\Lambda_0(U)\|^2+\|\boldsymbol{\beta}-\boldsymbol{\beta}_0\|^2+\|g(\mathbf{Z})-g_0(\mathbf{Z})\|^2 \\ &+\|\boldsymbol{\gamma}-\boldsymbol{\gamma}_0\|^2+\|h(\mathbf{Z})-h_0(\mathbf{Z})\|^2+|\zeta-\zeta_0|\} \\ &= d^2(\boldsymbol{\eta},\boldsymbol{\eta}_0)\,.\end{aligned}$$

On the other hand, according to van der Vaart (2000), Lemma 25.86,

$$\begin{aligned}\mathbb{E}[\boldsymbol{h}_{\boldsymbol{\eta}}(\boldsymbol{V})-\boldsymbol{h}_{\boldsymbol{\eta}_0}(\boldsymbol{V})]^2 &\gtrsim \mathbb{E}\{(\log\Lambda(U)-\log\Lambda_0(U))+(\boldsymbol{\beta}-\boldsymbol{\beta}_0)^\top\mathbf{X}+(g(\mathbf{Z})-g_0(\mathbf{Z})) \\ &+(\boldsymbol{\gamma}-\boldsymbol{\gamma}_0)^\top\mathbf{X}I_{\{E>\zeta_0\}}+(h(\mathbf{Z})-h_0(\mathbf{Z}))I_{\{E>\zeta_0\}}-(\boldsymbol{\gamma}^\top\mathbf{X}+h(\mathbf{Z}))I_{\{\zeta_0<E\leq\zeta\}}\}^2 \\ &\gtrsim \mathbb{E}\{(\|\Lambda(U)-\Lambda_0(U)\|^2+\|\boldsymbol{\beta}-\boldsymbol{\beta}_0\|^2+\|g(\mathbf{Z})-g_0(\mathbf{Z})\|^2 \\ &+\|\boldsymbol{\gamma}-\boldsymbol{\gamma}_0\|^2+\|h(\mathbf{Z})-h_0(\mathbf{Z})\|^2+|\zeta-\zeta_0|\} \\ &= d^2(\boldsymbol{\eta},\boldsymbol{\eta}_0)\,.\end{aligned}$$

The other side that $\zeta < \zeta_0$ shows the same results. Hence $\ell(\boldsymbol{\eta})-\ell(\boldsymbol{\eta}_0) \asymp -d^2(\boldsymbol{\eta},\boldsymbol{\eta}_0)$. □

Proof of Theorem 2

The proof is divided into two parts: we first establish the consistency that $d(\hat{\boldsymbol{\eta}}, \boldsymbol{\eta}_0) = o_P(1)$, then give a more precise result of the overall convergence rate, namely that $d(\hat{\boldsymbol{\eta}}, \boldsymbol{\eta}_0) = O_P(\alpha_n \log^2 n + n^{-u\nu})$.

We first demonstrate $d(\hat{\boldsymbol{\eta}}, \boldsymbol{\eta}_0) = o_P(1)$. Define $\mathcal{S}_D = \mathcal{F}_D \times \mathbb{R}^p_D \times \mathcal{G}_D \times \mathbb{R}^p_D \times \mathcal{G}_D \times \mathbb{R}_D$, and

$$\hat{\boldsymbol{\eta}}_D = (\hat{\Lambda}_D, \hat{\boldsymbol{\beta}}_D, \hat{g}_D, \hat{\boldsymbol{\gamma}}_D, \hat{h}_D, \hat{\zeta}_D) = \arg\max_{\boldsymbol{\eta} \in \mathcal{S}_D} \ell_n(\boldsymbol{\eta}) \,. \tag{3}$$

In a bounded set, the exponential functions and the logarithmic functions are Lipschitz continuous, it suffices to prove the class $\{m_{\boldsymbol{\eta}} : \boldsymbol{\eta} \in \mathcal{S}_D\}$ is a P-Glivenko-Cantalli class. By Theorem 2.7.11 in van der Vaart and Wellner (1996) and Lemma 9.18 in Kosorok (2008), the bracketing number of $\{m_{\boldsymbol{\eta}} : \boldsymbol{\eta} \in \mathcal{S}_D\}$ can be controlled by

$$\mathcal{N}_{[]}(\epsilon, \{m_{\boldsymbol{\eta}} : \boldsymbol{\eta} \in \mathcal{S}_D\}, d(\cdot,\cdot)) \le \mathcal{N}(\epsilon, \mathcal{S}_D, L^1(\mathbb{P})) \le \mathcal{N}_{[]}(\epsilon, \mathcal{S}_D, L^1(\mathbb{P}))\,,$$

so it only needs to show the bracketing number of $\mathcal{S}_D$ is finite. Since $\mathbb{R}^p_D$ and $\mathbb{R}_D$ are finite-dimensional and $\mathcal{F}_D$ is a class of monotone function, combining Lemma 6 in Zhong et al. (2022), Lemma 9.25 of Kosorok (2008) yields

$$\mathcal{N}(\epsilon, \mathcal{S}_D, L^1(\mathbb{P})) \le \mathcal{N}_{[]}(\epsilon, \mathcal{F}_D, \|\cdot\|_{L^2(\mathbb{P})}) \times \mathcal{N}^2_{[]}(\epsilon, \mathbb{R}^p_D, \|\cdot\|) \times \mathcal{N}^2_{[]}(\epsilon, \mathcal{G}_D, \|\cdot\|_{L^2(\mathbb{P})}) \times \mathcal{N}_{[]}(\epsilon, \mathbb{R}_D, \|\cdot\|)\,,$$

and Theorem 2.4.1 of van der Vaart and Wellner (1996) implies $\{m_{\boldsymbol{\eta}} : \boldsymbol{\eta} \in \mathcal{S}_D\}$ is a P-G-C class, thus

$$\sup_{\boldsymbol{\eta} \in \mathcal{S}_D} |\ell_n(\boldsymbol{\eta}) - \ell(\boldsymbol{\eta})| \xrightarrow{P} 0\,. \tag{4}$$

According to Lemma 1,

$$\sup_{\boldsymbol{\eta}: d(\boldsymbol{\eta}, \boldsymbol{\eta}_0) \ge \epsilon} \ell(\boldsymbol{\eta}) < \ell(\boldsymbol{\eta}_0)\,.$$

Let $g_{0,n} = \arg\min_{g \in \mathcal{G}(s,K,p,D/2)} \|g - g_0\|_{L^2([0,1]^r)}$ and $h_{0,n} = \arg\min_{h \in \mathcal{G}(s,K,p,D/2)} \|h - h_0\|_{L^2([0,1]^r)}$, according to the proof of Theorem 1 in Schmidt-Hieber (2020), $\|g_{0,n} - g_0\|_{L^2([0,1]^r)} = O(\alpha_n^2 \log^2 n)$ and $\|h_{0,n} - h_0\|_{L^2([0,1]^r)} = O(\alpha_n^2 \log^2 n)$. Define $\Lambda_{0,n} = \arg\max_{\Lambda \in \mathcal{F}_{D/2}} \|\Lambda - \Lambda_0\|_{L^2([0,1]^r)}$, Lu and Zhang (2007) have proved that $\|\Lambda_{0,n} - \Lambda_0\|_{L^2([0,1]^r)} = O(n^{-u\nu})$ for $(2u+1)^{-1} < \nu < (2u)^{-1}$. Combining (4) with Lemma 2, the law of large numbers yields

$$\begin{aligned}
&|\ell_n(\Lambda_{0,n}, \boldsymbol{\beta}_0, g_{0,n}, \boldsymbol{\gamma}_0, h_{0,n}, \zeta_0) - \ell_n(\Lambda_0, \boldsymbol{\beta}_0, g_0, \boldsymbol{\gamma}_0, h_0, \zeta_0)| \\
\le& |\ell_n(\Lambda_{0,n}, \boldsymbol{\beta}_0, g_{0,n}, \boldsymbol{\gamma}_0, h_{0,n}, \zeta_0) - \ell(\Lambda_{0,n}, \boldsymbol{\beta}_0, g_{0,n}, \boldsymbol{\gamma}_0, h_{0,n}, \zeta_0)| \\
&+ |\ell(\Lambda_{0,n}, \boldsymbol{\beta}_0, g_{0,n}, \boldsymbol{\gamma}_0, h_{0,n}, \zeta_0) - \ell(\Lambda_0, \boldsymbol{\beta}_0, g_{0,n}, \boldsymbol{\gamma}_0, h_{0,n}, \zeta_0)| \\
&+ |\ell(\Lambda_0, \boldsymbol{\beta}_0, g_{0,n}, \boldsymbol{\gamma}_0, h_{0,n}, \zeta_0) - \ell(\Lambda_0, \boldsymbol{\beta}_0, g_0, \boldsymbol{\gamma}_0, h_{0,n}, \zeta_0)| \\
&+ |\ell(\Lambda_0, \boldsymbol{\beta}_0, g_0, \boldsymbol{\gamma}_0, h_{0,n}, \zeta_0) - \ell(\Lambda_0, \boldsymbol{\beta}_0, g_0, \boldsymbol{\gamma}_0, h_0, \zeta_0)| \\
&+ |\ell(\Lambda_0, \boldsymbol{\beta}_0, g_0, \boldsymbol{\gamma}_0, h_0, \zeta_0) - \ell_n(\Lambda_0, \boldsymbol{\beta}_0, g_0, \boldsymbol{\gamma}_0, h_0, \zeta_0)| \\
=& o_P(1)\,,
\end{aligned}$$

which implies

$$\ell_n(\hat{\boldsymbol{\eta}}_D) \ge \ell_n(\boldsymbol{\eta}_0) - o_P(1)\,. \tag{5}$$

Combining (3), (4) and (5), Theorem 5.7 of van der Vaart (2000) yields $d(\hat{\boldsymbol{\eta}}_D, \boldsymbol{\eta}_0) \xrightarrow{P} 0$.

In the second part, we turn to derive the overall convergence rate. Let

$$\mathcal{A}_\delta = \{\boldsymbol{\eta} \in \mathcal{S}_D : \delta/2 \le d(\boldsymbol{\eta}, \boldsymbol{\eta}_0) \le \delta\}\,,$$

according to Lemma 2, we have

$$\sup_{\boldsymbol{\eta} \in \mathcal{A}_\delta} \{\ell(\boldsymbol{\eta}) - \ell(\boldsymbol{\eta}_0)\} \lesssim -\delta^2\,.$$

Define $\phi_n(\delta) = \delta\sqrt{s \log \frac{L}{\delta}} + \frac{s}{\sqrt{n}} \log \frac{L}{\delta} + \sqrt{n}\{\alpha_n \log^2 n + n^{-u\nu}\}^2$, by Lemma 1 and its proof we obtain

$$\mathbb{E}^* \sup_{\boldsymbol{\eta} \in \mathcal{A}_\delta} |(\ell_n - \ell)(\boldsymbol{\eta}) - (\ell_n - \ell)(\boldsymbol{\eta}_0)| \lesssim \frac{\phi_n(\delta)}{\sqrt{n}}\,. \tag{6}$$

It is clear that for any $a > 2$, $\phi_n(\delta)/\delta^a$ is decreasing. Let $r_n = \alpha_n \log^2 n + n^{-u\nu}$,

$$r_n^{-2}\phi_n(r_n) = \left\{ r_n \sqrt{s \log \frac{L}{r_n}} + \frac{s}{\sqrt{n}} \log \frac{L}{r_n} + \sqrt{n} r_n^2 \right\} r_n^{-2} \le R\sqrt{n}\,, \tag{7}$$

where $0 < R < +\infty$ denotes some constant. According to (6) and (7), denote $\boldsymbol{\eta}_{0,n} = (\Lambda_{0,n}, \boldsymbol{\beta}_0, g_{0,n}, \boldsymbol{\gamma}_0, h_{0,n}, \zeta_0)$, we have

$$
\begin{aligned}
&|\ell_n(\Lambda_{0,n}, \boldsymbol{\beta}_0, g_{0,n}, \boldsymbol{\gamma}_0, h_{0,n}, \zeta_0) - \ell_n(\Lambda_0, \boldsymbol{\beta}_0, g_0, \boldsymbol{\gamma}_0, h_0, \zeta_0)| \\
\lesssim & O_P(n^{-1/2}\phi_n(r_n)) + |\ell(\Lambda_{0,n}, \boldsymbol{\beta}_0, g_{0,n}, \boldsymbol{\gamma}_0, h_{0,n}, \zeta_0) - \ell(\Lambda_0, \boldsymbol{\beta}_0, g_0, \boldsymbol{\gamma}_0, h_0, \zeta_0)| \\
\lesssim & O_P(n^{-1/2}\phi_n(r_n)) + \|\Lambda_{0,n} - \Lambda_0\|^2_{L^2([0,T])} + \|g_{0,n} - g_0\|^2_{L^2([0,1]^r)} + \|h_{0,n} - h_0\|^2_{L^2([0,1]^r)} \\
= & O_P(r_n^2)\,.
\end{aligned}
$$

By the definition of $\hat{\boldsymbol{\eta}}_D$, there exists

$$
\ell_n(\hat{\boldsymbol{\eta}}_D) \geq \ell_n(\boldsymbol{\eta}_{0,n}) = \ell_n(\boldsymbol{\eta}_0) - O_P(r_n^2)\,. \tag{8}
$$

Finally, combining (6), (7) and (8), Theorem 3.4.1 of van der Vaart and Wellner (1996) leads to

$$
r_n^{-1} d(\hat{\boldsymbol{\eta}}_D, \boldsymbol{\eta}_0) = O_P(1)\,,
$$

which demonstrates that $d(\hat{\boldsymbol{\eta}}_n, \boldsymbol{\eta}_0) = O_P(\alpha_n \log^2 n + n^{-u\nu})$. □

Proof of Lemma 3

Let $U_\epsilon(\zeta_0, \boldsymbol{\theta}_0) = \{(\zeta, \boldsymbol{\theta}) : A < n^{1/2}(|\zeta - \zeta_0| + \|\boldsymbol{\theta} - \boldsymbol{\theta}_0\|^2)^{1/2} \leq n^{1/2}\epsilon\}$ and $V_\epsilon(\zeta_0, \boldsymbol{\theta}_0) = \{(\zeta, \boldsymbol{\theta}) : \left(|\zeta - \zeta_0| + \|\boldsymbol{\theta} - \boldsymbol{\theta}_0\|^2\right)^{1/2} < \epsilon\}$. According to Theorem 2 and the condition of Lemma 3, for any $r, u \in (0,1)$ and n large enough, $\mathbb{P}((\xi, \boldsymbol{\theta}) \in V_\epsilon(\zeta_0, \boldsymbol{\theta}_0)) > 1 - r$ and

$$
\mathbb{P}\left(\sup_{(\zeta,\boldsymbol{\theta})} \ell_n(\zeta, \boldsymbol{\theta}|g, h, \Lambda) - \sup_{(\zeta,\boldsymbol{\theta})} \ell_n(\zeta, \boldsymbol{\theta}|g_0, h_0, \Lambda_0) \geq 0\right) \leq u\,,
$$

thus

$$
\begin{aligned}
&\mathbb{P}\left(n^{1/2}(|\hat{\zeta}_n - \zeta_0| + \|\hat{\boldsymbol{\theta}}_n - \boldsymbol{\theta}_0\|^2)^{1/2} > A\right) \\
=&\mathbb{P}\left((\hat{\zeta}_n, \hat{\boldsymbol{\theta}}_n) \in U_\epsilon(\zeta_0, \boldsymbol{\theta}_0)\right) + \mathbb{P}\left((\hat{\zeta}_n, \hat{\boldsymbol{\theta}}_n) \in V_\epsilon^C(\zeta_0, \boldsymbol{\theta}_0)\right) \\
\leq&\mathbb{P}\left(\sup_{(\zeta,\boldsymbol{\theta})} \ell_n(\zeta, \boldsymbol{\theta}|g, h, \Lambda) \geq \ell_n(\zeta_0, \boldsymbol{\theta}_0|g_0, h_0, \Lambda_0)\right) + r \\
\leq&\mathbb{P}\left(\sup_{(\zeta,\boldsymbol{\theta})} G_n(\zeta, \boldsymbol{\theta}) \geq 0\right) + u + r\,,
\end{aligned}
$$

where $G_n(\zeta, \boldsymbol{\theta}) = n\ell_n(\zeta, \boldsymbol{\theta}|g_0, h_0, \Lambda_0) - n\ell_n(\zeta_0, \boldsymbol{\theta}_0|g_0, h_0, \Lambda_0)$ and

$$
\begin{aligned}
&\mathbb{P}\left(\sup_{(\zeta,\boldsymbol{\theta})} \ell_n(\zeta, \boldsymbol{\theta}|g, h, \Lambda) \geq \ell_n(\zeta_0, \boldsymbol{\theta}_0|g, h, \Lambda_0)\right) \\
=&\mathbb{P}\Bigg(\sup_{(\zeta,\boldsymbol{\theta})} \ell_n(\zeta, \boldsymbol{\theta}|g, h, \Lambda) - \sup_{(\zeta,\boldsymbol{\theta})} \ell_n(\zeta, \boldsymbol{\theta}|g_0, h_0, \Lambda_0) \\
&\qquad + \sup_{(\zeta,\boldsymbol{\theta})} \ell_n(\zeta, \boldsymbol{\theta}|g_0, h_0, \Lambda_0) - \ell_n(\zeta_0, \boldsymbol{\theta}_0|g, h, \Lambda_0) \geq 0\Bigg) \\
\leq&\mathbb{P}\left(\sup_{(\zeta,\boldsymbol{\theta})} \ell_n(\zeta, \boldsymbol{\theta}|g_0, h_0, \Lambda_0) - \ell_n(\zeta_0, \boldsymbol{\theta}_0|g_0, h_0, \Lambda_0)\right) + u\,.
\end{aligned}
$$

Let $G(\zeta, \boldsymbol{\theta})$ be the expectation of $G_n(\zeta, \boldsymbol{\theta})$, G's Taylor expansion states

$$
G(\zeta, \boldsymbol{\theta}) = \frac{\partial}{\partial\zeta}\dot{G}_\zeta(\zeta, \boldsymbol{\theta})(\zeta - \zeta_0) - (\boldsymbol{\theta} - \boldsymbol{\theta}_0)^\top I(\zeta^*, \boldsymbol{\theta}^*)(\boldsymbol{\theta} - \boldsymbol{\theta}_0) + o(1)\,,
$$

and by linearization the first term is negative and the information $I(\zeta_0, \boldsymbol{\theta}_0)$ is positive definite, thus there exists $k_0 > 0$ such that $G(\zeta, \boldsymbol{\theta}) \leq -k_0(|\hat{\zeta}_n - \zeta_0| + \|\hat{\boldsymbol{\theta}}_n - \boldsymbol{\theta}_0\|^2)^{1/2}$. Split the set $U_\epsilon(\zeta_0, \boldsymbol{\theta}_0)$ by sets

$$
H_{n,j} := \left\{(\zeta, \boldsymbol{\theta}) : 2^j \leq n^{1/2}(|\hat{\zeta}_n - \zeta_0| + \|\hat{\boldsymbol{\theta}}_n - \boldsymbol{\theta}_0\|^2)^{1/2} < 2^{j+1}\right\}
$$

then for any $\tilde{\epsilon} > 0$, there exists $k \geq 0$ such that

$$
\mathbb{E} \sup_{(\zeta,\boldsymbol{\theta}) \in V_\epsilon(\zeta_0, \boldsymbol{\theta}_0)} |n^{1/2}\{G_n(\zeta, \boldsymbol{\theta}) - G(\zeta, \boldsymbol{\theta})\}| \leq k\tilde{\epsilon}
$$

for $n \to \infty$. By Chebyshev's inequality and Lemma 3 of Pons (2003),

$$\begin{aligned}
&\limsup_{n\to\infty} \sum_{j:2^j>A} \mathbb{P}\left\{\sup_{H_{n,j}} n^{1/2}(G_n(\zeta,\boldsymbol{\theta}) - G(\zeta,\boldsymbol{\theta})) \geq n^{-1/2}2^{2j}k_0\right\} \\
\leq &\limsup_{n\to\infty} \sum_{j:2^j>A} \frac{\mathbb{E}\left\{\sup_{H_{n,j}} n(G_n(\zeta,\boldsymbol{\theta}) - G(\zeta,\boldsymbol{\theta}))\right\}^2}{2^{4j}k_0^2} \\
\leq &\sup_{\tilde{\epsilon},k_0}\left(\frac{k^2}{k_0^2}\right)\sum_{j:2^j>A}\frac{1}{2^{2j-2}} \to 0
\end{aligned}$$

as $A \to +\infty$, hence $\lim_{A\to\infty}\limsup_{n\to\infty}\mathbb{P}\left(n^{1/2}(|\hat{\zeta}_n - \zeta_0| + \|\hat{\boldsymbol{\theta}}_n - \boldsymbol{\theta}_0\|^2)^{1/2} > A\right) = 0$, which yields the conclusion. □

Proof of Theorem 3

The log-likelihood function with plug-in estimator $\ell_n(\hat{\boldsymbol{\eta}}_n)$ has the following decomposition

$$\ell_n(\hat{\boldsymbol{\eta}}_n) = \{\ell_n(\hat{\boldsymbol{\eta}}_n) - \ell_n(\hat{\boldsymbol{\xi}}_n,\zeta_0)\} + \ell_n(\hat{\boldsymbol{\xi}}_n,\zeta_0)\,, \tag{9}$$

the second term of this decomposition is the log-likelihood function defined in Theorem 3, where ζ_0 is known to be the real value. Denote $G(x) = \log(1-e^x)$, the first term of (9) can be written as

$$\begin{aligned}
\ell_n(\hat{\boldsymbol{\eta}}_n) - \ell_n(\hat{\boldsymbol{\xi}}_n,\zeta_0) = &\frac{1}{n}\sum_{i=1}^n\int_{l_c}^{u_c}\boldsymbol{m}(t;\hat{\boldsymbol{\xi}}_n,\hat{\zeta}_n) - \boldsymbol{m}(t;\hat{\boldsymbol{\xi}}_n,\zeta_0)dN_{1i}(t) \\
&+\frac{1}{n}\sum_{i=1}^n\int_{l_c}^{u_c}G(\boldsymbol{m}(t;\hat{\boldsymbol{\xi}}_n,\hat{\zeta}_n)) - G(\boldsymbol{m}(t;\hat{\boldsymbol{\xi}}_n,\zeta_0))dN_{2i}(t)\,.
\end{aligned}$$

Let $\tilde{I}(\zeta) = I_{\{\zeta_0<E\leq\zeta\}} - I_{\{\zeta<E\leq\zeta_0\}}$, with some algebra, the first term is

$$\begin{aligned}
&\boldsymbol{m}(t;\hat{\boldsymbol{\xi}}_n,\hat{\zeta}_n) - \boldsymbol{m}(t;\hat{\boldsymbol{\xi}}_n,\zeta_0) \\
=&-\Lambda_n(t)\exp\left\{\hat{\boldsymbol{\beta}}_n^\top\mathbf{X} + \hat{g}_n(\mathbf{Z})\right\}\exp\left\{\{\hat{\boldsymbol{\gamma}}_n^\top\mathbf{X} + \hat{h}_n(\mathbf{Z})\}(I_{\{E>\hat{\zeta}_n\}} - I_{\{E>\zeta_0\}})\right\} \\
=&\mathcal{M}(t;\hat{\boldsymbol{\xi}}_n)\tilde{I}(\hat{\zeta}_n) \\
=&\mathcal{M}(t;\boldsymbol{\xi}_0)\tilde{I}(\hat{\zeta}_n) + o_P(1)\,.
\end{aligned}\tag{10}$$

For the second term in (9), if z denotes some variable that only takes value from 0 and 1 (such as indicator function), there exists

$$f(z) = f(0) + (f(1) - f(0))z\,,$$

thus if $\hat{\zeta}_n < \zeta_0$,

$$\begin{aligned}
&G(\boldsymbol{m}(t;\hat{\boldsymbol{\xi}}_n,\hat{\zeta}_n)) - G(\boldsymbol{m}(t;\hat{\boldsymbol{\xi}}_n,\zeta_0)) \\
=&\log\left(\frac{1-\exp\left\{-\hat{\Lambda}_n(t)\exp\{\hat{\boldsymbol{\beta}}_n^\top\mathbf{X} + \hat{g}_n(\mathbf{Z}) + \{\hat{\boldsymbol{\gamma}}_n^\top\mathbf{X} + \hat{h}_n(\mathbf{Z})\}(1 + I_{\{E>\zeta_0\}})\}\right\}}{1-\exp\left\{-\hat{\Lambda}_n(t)\exp\{\hat{\boldsymbol{\beta}}_n^\top\mathbf{X} + \hat{g}_n(\mathbf{Z}) + \{\hat{\boldsymbol{\gamma}}_n^\top\mathbf{X} + \hat{h}_n(\mathbf{Z})\}I_{\{E>\zeta_0\}}\}\right\}}\right)I_{\{\hat{\zeta}_n<E\leq\zeta_0\}} \\
=&\mathcal{M}^-(t;\boldsymbol{\xi}_0)I_{\{\hat{\zeta}_n<E\leq\zeta_0\}} + o_P(1)\,.
\end{aligned}\tag{11}$$

Similarly, if $\hat{\zeta}_n > \zeta_0$, we have

$$G(\boldsymbol{m}(t;\hat{\boldsymbol{\xi}}_n,\hat{\zeta}_n)) - G(\boldsymbol{m}(t;\hat{\boldsymbol{\xi}}_n,\zeta_0)) = \mathcal{M}^+(t,\boldsymbol{\xi}_0)I_{\{\zeta_0<E\leq\hat{\zeta}_n\}} + o_P(1)\,. \tag{12}$$

Combining (10), (11) and (12) for n subjects, let

$$\begin{aligned}
Q_n(\boldsymbol{\xi}_0,\hat{\zeta}_n) = &\sum_{i=1}^n\int_{l_c}^{u_c}\mathcal{M}(t;\boldsymbol{\xi}_0)\tilde{I}(\hat{\zeta}_n)dN_{1i}(t) \\
&+\sum_{i=1}^n\int_{l_c}^{u_c}\left\{\mathcal{M}^+(t;\boldsymbol{\xi}_0)I_{\{\zeta_0<E\leq\hat{\zeta}_n\}} - \mathcal{M}^-(t;\boldsymbol{\xi}_0)I_{\{\hat{\zeta}_n<E\leq\zeta_0\}}\right\}dN_{2i}(t)\,,
\end{aligned}$$

it can be concluded that

$$\ell_n(\hat{\boldsymbol{\eta}}_n) = n^{-1}Q_n(\boldsymbol{\xi}_0, \hat{\zeta}_n) + \ell_n(\hat{\boldsymbol{\xi}}_n, \zeta_0) + o_P(1)\,,$$

which yields the asymptotic independence between $\hat{\boldsymbol{\xi}}_n$ and $\hat{\zeta}_n$. □

Proof of Theorem 4

Denote $\boldsymbol{\theta} = (\boldsymbol{\beta}^\top, \boldsymbol{\gamma}^\top)^\top \in \mathbb{R}^{2p}$, $\mathbf{Y} = (\Delta, \boldsymbol{V})$, let $\tilde{\mathbf{X}}(\zeta_0) = (\mathbf{X}^\top, \mathbf{X}^\top I_{\{E>\zeta_0\}})^\top$ and $\tilde{K}(\mathbf{Z};\zeta_0) = g(\mathbf{Z}) + h(\mathbf{Z})I_{\{E>\zeta_0\}}$, for a given ζ_0, $\boldsymbol{h}_{\boldsymbol{\eta}}(\boldsymbol{V})$ can be reorganized as

$$\boldsymbol{h}_{\boldsymbol{\eta}}(\boldsymbol{V}) = \exp\{\log \Lambda(U) + \boldsymbol{\theta}^\top \tilde{\mathbf{X}}(\zeta_0) + \tilde{K}(\mathbf{Z}, \zeta_0)\}\,.$$

The efficient score function with respect to $\boldsymbol{\theta}_0$ is

$$U(\boldsymbol{\theta}_0;\zeta_0) = \ell^*_{\boldsymbol{\theta}}(\mathbf{Y};\boldsymbol{\eta}_0) := \dot{\ell}_{\boldsymbol{\theta}}(\boldsymbol{\eta}_0) - \prod_{\Lambda_0,\tilde{K}_0} (\dot{\ell}_{\boldsymbol{\theta}}(\boldsymbol{\eta}_0) \mid \dot{\mathcal{P}}_1 + \dot{\mathcal{P}}_2)\,,$$

where $\dot{\mathcal{P}}_1 = \{\dot{\ell}_\Lambda(\boldsymbol{\eta}_0)[\boldsymbol{a}], \boldsymbol{a} \in \bar{\mathbb{T}}_{\Lambda_0}\}$, $\dot{\mathcal{P}}_2 = \{\dot{\ell}_{\tilde{K}}(\boldsymbol{\eta}_0)[\boldsymbol{b}], \boldsymbol{b} \in \bar{\mathbb{T}}_{\tilde{K}_0}\}$ and $\prod_{\Lambda_0,\tilde{K}_0}(\dot{\ell}_{\boldsymbol{\theta}}(\boldsymbol{\xi}_0,\zeta) \mid \dot{\mathcal{P}}_1 + \dot{\mathcal{P}}_2)$ denotes the projection of $\dot{\ell}_{\boldsymbol{\theta}}(\boldsymbol{\eta}_0)$ onto the subspace $\dot{\mathcal{P}}_1 + \dot{\mathcal{P}}_2$. According to Subsection 21.1.4 in Kosorok (2008), the least favorable direction $(\boldsymbol{a}_*, \boldsymbol{b}_*) \in \bar{\mathbb{T}}^{2p}_{\Lambda_0} \times \bar{\mathbb{T}}^{2p}_{\tilde{K}_0}$ satisfies

$$\mathbb{E}\left\{\left(\dot{\ell}_{\boldsymbol{\theta}}(\boldsymbol{\eta}_0) - \dot{\ell}_\Lambda(\boldsymbol{\eta}_0)[\boldsymbol{a}_*] - \dot{\ell}_{\tilde{K}}(\boldsymbol{\eta}_0)[\boldsymbol{b}_*]\right)\dot{\ell}_\Lambda(\boldsymbol{\eta}_0)[\boldsymbol{a}]\right\} = 0, \text{ for all } \boldsymbol{a} \in \bar{\mathbb{T}}_{\Lambda_0}\,,$$

$$\mathbb{E}\left\{\left(\dot{\ell}_{\boldsymbol{\theta}}(\boldsymbol{\eta}_0) - \dot{\ell}_\Lambda(\boldsymbol{\eta}_0)[\boldsymbol{a}_*] - \dot{\ell}_{\tilde{K}}(\boldsymbol{\eta}_0)[\boldsymbol{b}_*]\right)\dot{\ell}_{\tilde{K}}(\boldsymbol{\eta}_0)[\boldsymbol{b}]\right\} = 0, \text{ for all } \boldsymbol{b} \in \bar{\mathbb{T}}_{\tilde{K}_0}\,,$$

thus $\prod_{\Lambda_0,\tilde{K}_0}(\dot{\ell}_{\boldsymbol{\theta}}(\boldsymbol{\eta}_0) \mid \dot{\mathcal{P}}_1 + \dot{\mathcal{P}}_2) = \dot{\ell}_\Lambda(\boldsymbol{\eta}_0)[\boldsymbol{a}_*] + \dot{\ell}_{\tilde{K}}(\boldsymbol{\eta}_0)[\boldsymbol{b}_*]$. The least favorable direction can be found by solving the equations

$$\mathbb{E}\left\{\left(\dot{\ell}_{\boldsymbol{\theta}}(\boldsymbol{\eta}_0) - \dot{\ell}_\Lambda(\boldsymbol{\eta}_0)[\boldsymbol{a}_*] - \dot{\ell}_{\tilde{K}}(\boldsymbol{\eta}_0)[\boldsymbol{b}_*]\right) \mid U = u\right\} = 0$$

and

$$\mathbb{E}\left\{\left(\dot{\ell}_{\boldsymbol{\theta}}(\boldsymbol{\eta}_0) - \dot{\ell}_\Lambda(\boldsymbol{\eta}_0)[\boldsymbol{a}_*] - \dot{\ell}_{\tilde{K}}(\boldsymbol{\eta}_0)[\boldsymbol{b}_*]\right) \mid \mathbf{Z} = \mathbf{z}\right\} = 0\,.$$

This indicates $(\boldsymbol{a}_*^\top, \boldsymbol{b}_*^\top)^\top$ minimizes

$$\begin{aligned}&\mathbb{E}\left\{\left[\dot{\ell}_{\boldsymbol{\theta}}(\boldsymbol{\eta}_0) - \dot{\ell}_\Lambda(\boldsymbol{\eta}_0)[\boldsymbol{a}] - \dot{\ell}_{\tilde{K}}(\boldsymbol{\eta}_0)[\boldsymbol{b}]\right] \odot \left[\dot{\ell}_{\boldsymbol{\theta}}(\boldsymbol{\eta}_0) - \dot{\ell}_\Lambda(\boldsymbol{\eta}_0)[\boldsymbol{a}] - \dot{\ell}_{\tilde{K}}(\boldsymbol{\eta}_0)[\boldsymbol{b}]\right]\right\}\\ =&\mathbb{E}\left\{\mathcal{Q}^2(\mathbf{Y};\boldsymbol{\xi}_0,\zeta_0)[(\tilde{\mathbf{X}}(\zeta_0) - \boldsymbol{a}(U) - \boldsymbol{b}(\mathbf{Z})) \odot (\tilde{\mathbf{X}}(\zeta_0) - \boldsymbol{a}(U) - \boldsymbol{b}(\mathbf{Z}))]\right\}\,.\end{aligned}$$

By (C3)-(C7) and Bickle et al. (1993), the minimizer is well-defined and unique. Therefore, the efficient score function is

$$U(\boldsymbol{\theta}_0;\zeta_0) = \ell^*_{\boldsymbol{\theta}}(Y,\boldsymbol{\eta}_0) = \{\tilde{\mathbf{X}}(\zeta_0) - \boldsymbol{a}_*(U) - \boldsymbol{b}_*(\mathbf{Z})\}\mathcal{Q}(\mathbf{Y};\boldsymbol{\xi}_0,\zeta_0)\,,$$

and the information matrix is

$$I(\boldsymbol{\theta}_0;\zeta_0) = \mathbb{E}(U(\boldsymbol{\theta}_0;\zeta_0)^{\otimes 2}) = \mathbb{E}\left\{\mathcal{Q}^2(\mathbf{Y};\boldsymbol{\xi}_0,\zeta_0)\{\tilde{\mathbf{X}}(\zeta_0) - \boldsymbol{a}_*(U) - \boldsymbol{b}_*(\mathbf{Z})\}^{\otimes 2}\right\}\,.$$

□

Proof of Theorem 5

Theorem 3 implies that $\hat{\zeta}_n$ is asymptotically independent of $\hat{\boldsymbol{\xi}}_n$. For $\ell(\hat{\boldsymbol{\xi}}_n,\zeta_0)$, the expectation of $\ell_n(\hat{\boldsymbol{\xi}}_n,\zeta_0)$ defined in Theorem 3, by the definition of $\hat{\boldsymbol{\xi}}_n$,

$$\mathbb{P}_n\ell^*_{\boldsymbol{\theta}}(\mathbf{Y};\hat{\boldsymbol{\xi}}_n,\zeta_0) = 0\,. \tag{13}$$

By Theorem 2, we have

$$(\mathbb{P}_n - \mathbb{P})\left\{\ell^*_{\boldsymbol{\theta}}(\mathbf{Y};\hat{\boldsymbol{\xi}}_n,\zeta_0) - \ell^*_{\boldsymbol{\theta}}(\mathbf{Y};\boldsymbol{\eta}_0)\right\} = o_P(n^{-1/2})\,. \tag{14}$$

Using Taylor expansion, there exists

$$\mathbb{P}\left\{\ell^*_{\boldsymbol{\theta}}(\mathbf{Y};\hat{\boldsymbol{\xi}}_n,\zeta_0) - \ell^*_{\boldsymbol{\theta}}(\mathbf{Y};\boldsymbol{\eta}_0)\right\} = -\mathbb{P}\left\{\ell^*_{\boldsymbol{\theta}}(\mathbf{Y};\boldsymbol{\eta}_0)^{\otimes 2}\right\}(\hat{\boldsymbol{\theta}}_n - \boldsymbol{\theta}_0) + O(d^2(\hat{\boldsymbol{\xi}}_n,\boldsymbol{\xi}_0))\,. \tag{15}$$

Similar to Theorem 2, if ζ_0 is fixed, $d^2(\hat{\boldsymbol{\xi}}_n, \boldsymbol{\xi}_0) = O_P(\alpha_n^2 \log^2 n + n^{-u\nu})$ and $(2u+1)^{-1} < \nu < (2u)^{-1}$ with $u \geq 1$, and we have assumed $n\alpha_n^4 \to \infty$ as $n \to \infty$. Hence $O(d^2(\hat{\boldsymbol{\xi}}_n, \boldsymbol{\xi}_0) = o_P(n^{-1/2})$. Combining (13), (14) and (15), it suffices that

$$\sqrt{n}(\hat{\boldsymbol{\theta}}_n - \boldsymbol{\theta}_0) = \sqrt{n} I^{-1}(\boldsymbol{\theta}_0; \zeta_0) \sum_{i=1}^{n} \ell^*_{\boldsymbol{\theta}}(\mathbf{Y}_i; \boldsymbol{\eta}_0) + o_P(1) \,,$$

and the central limit theorem yields $\sqrt{n}(\hat{\boldsymbol{\theta}}_n - \boldsymbol{\theta}_0) \xrightarrow{d} N(\mathbf{0}, I^{-1}(\boldsymbol{\theta}_0; \zeta_0))$.

Then we turn to the asymptotic properties of $\hat{\zeta}_n$, and it needs to verify $\tilde{Q}_n(u) \xrightarrow{d} \tilde{Q}(u)$. Denote $i = \sqrt{-1}$, the process

$$\begin{aligned} \tilde{Q}_n^+(v) &= I_{\{v>0\}} \sum_{i=1}^{n} \xi_i^+ I_{\{\zeta_0 + n^{-1}v < E_i \leq \zeta_0\}} \\ &= I_{\{v>0\}} \sum_{i=1}^{\mathbf{v}_n^+} \xi_i^+ \,, \end{aligned}$$

where $\mathbf{v}_n^+ = \sum_{i=1}^n I_{\{\zeta_0 < E_i \leq \zeta_0 + n^{-1}v\}}$ follows a Binomial distribution $B(n, p_n^+)$, $p_n^+ = \mathbb{P}(\zeta_0 < E_i \leq \zeta_0 + n^{-1}v)$ such that $0 < np_n^+ \to vh_3(\zeta_0) < +\infty$. Symmetrically, for $v < 0$, let $\mathbf{v}_n^- = \sum_{i=1}^n I_{\{\zeta_0 + n^{-1}v < E_i \leq \zeta_0\}} \sim B(n, p_n^-)$, where $p_n^- = \mathbb{P}(\zeta_0 + n^{-1}v < E_i \leq \zeta_0)$ and $np_n^- \to -vh_3(\zeta_0) < +\infty$. Let $(V_k^+)_{k\geq 1}$ and $(V_k^-)_{k \geq 1}$ be independent sequences of i.i.d. random variables with the characteristic functions

$$\begin{aligned} \phi^+(t) &= \mathbb{E}(\exp\{itV_k^+\}) \\ &= \mathbb{E}\left\{\exp\left\{it((1-\Delta)\mathcal{M}(U, \boldsymbol{\xi}_0) + \Delta\mathcal{M}^+(U, \boldsymbol{\xi}_0)) \mid E = \zeta_0^+\right\}\right\} \,, \end{aligned}$$

$$\begin{aligned} \phi^-(t) &= \mathbb{E}(\exp\{itV_k^-\}) \\ &= \mathbb{E}\left\{\exp\left\{it((1-\Delta)\mathcal{M}(U, \boldsymbol{\xi}_0) + \Delta\mathcal{M}^-(U, \boldsymbol{\xi}_0)) \mid E = \zeta_0\right\}\right\} \end{aligned}$$

and let $V_0^+ = V_0^- = 0$. In addition, $(V_k^+)_{k\geq 1}$ and $(V_k^-)_{k\geq 1}$ are independent of $\mathbf{v}_n^+$ and $\mathbf{v}_n^-$. The process $\tilde{Q}_n^+$ and $\tilde{Q}_n^-$ show the same distribution with

$$\tilde{Q}_n^+(v) \stackrel{d}{=} I_{\{v>0\}} \sum_{0 \leq k \leq \mathbf{v}_n^+(v)} V_k^+ \,, \; \tilde{Q}_n^-(v) \stackrel{d}{=} I_{\{v<0\}} \sum_{0 \leq k \leq \mathbf{v}_n^-(v)} V_k^- \,.$$

Since $|vh_3(\zeta_0)| < \infty$, $\mathbf{v}_n^+$ and $\mathbf{v}_n^-$ converge to Poisson distributions with hazard $vh_3(\zeta_0)$ and $-vh_3(\zeta_0)$ respectively. Let $\mathbf{v}^+(\cdot)$ and $\mathbf{v}^-(\cdot)$ be the real jump processes such that $\mathbf{v}^+(0) = 0$ and $\mathbf{v}^-(0) = 0$, $\mathbf{v}^+(v)$ is a Poisson variable with parameter $vh_3(\zeta_0)$ on $\mathbb{R}^+$ and $\mathbf{v}^-(v)$ is a Poisson variable with parameter $-vh_3(\zeta_0)$ on $\mathbb{R}^-$. The right-continuous jump process $\tilde{Q} = \tilde{Q}^+ - \tilde{Q}^-$ where

$$\tilde{Q}^+(v) \stackrel{d}{=} I_{\{v>0\}} \sum_{0 \leq k \leq \mathbf{v}^+(v)} V_k^+ \,, \; \tilde{Q}^-(v) \stackrel{d}{=} I_{\{v<0\}} \sum_{0 \leq k \leq \mathbf{v}^-(v)} V_k^- \,.$$

We demonstrate $\tilde{Q}_n \xrightarrow{d} \tilde{Q}$ by illustrating the convergence of their characteristic functions. For $\tilde{Q}_n^+$ and $\tilde{Q}^+$ and $u \in \mathbb{R}^+$, let $\mu = h_3(\zeta_0)$, using the independence assumption of $(V_k^+)_{k\geq 0}$, there

exist

$$\begin{aligned}
\phi^+_{n,v}(t) &= \mathbb{E}\left(\mathbb{E}\left(e^{it\tilde{Q}^+_n(v)} \mid \mathbf{v}^+_n(v)\right)\right) \\
&= \mathbb{E}\left(\mathbb{E}\left(e^{it\sum_{0\le k\le \mathbf{v}^+_n(v)} V^+_k} \mid \mathbf{v}^+_n(v)\right)\right) \\
&= \mathbb{E}\left(e^{it\sum_{k=0}^n V^+_k I_{\{\zeta_0<E_k\le\zeta_0+n^{-1}v\}}}\right) \\
&= \mathbb{E}\prod_{k=0}^n \left(e^{itV^+_k I_{\{\zeta_0<E_k\le\zeta_0+n^{-1}v\}}}\right) \\
&= \mathbb{E}\prod_{k=0}^n \left(1+(e^{itV^+_k}-1)I_{\{\zeta_0<E_k\le\zeta_0+n^{-1}v\}}\right) \\
&= \mathbb{E}\prod_{k=0}^n \left(1+\frac{\mu u(e^{itV^+_k}-1)}{n}\right) \\
&= \mathbb{E}\left(1+\frac{\mu v(e^{itV^+_1}-1)}{n}\right)^{n+1} \\
&\to \mathbb{E}\exp\{v\mu(e^{itV^+_1}-1)\}
\end{aligned}$$

and

$$\begin{aligned}
\phi^+_v(t) &= \mathbb{E}\left[\mathbb{E}\left\{e^{it\tilde{Q}^+(v)} \mid \mathbf{v}^+(v)\right\}\right] \\
&= \sum_{j\ge 0}\frac{(\mu v)^j}{j!}e^{-\mu v}\mathbb{E}e^{it\sum_{0\le k\le j} V^+_k} \\
&= e^{-\mu v}\sum_{j\ge 0}\frac{(\mu v\phi^+(t))^j}{j!} \\
&= e^{-\mu v}\mathbb{E}e^{\mu v e^{itV^+_1}} \\
&= \mathbb{E}\exp\{v\mu(e^{itV^+_1}-1)\},
\end{aligned}$$

which implies $\tilde{Q}^+_n \xrightarrow{d} \tilde{Q}^+$, similarly $\tilde{Q}^-_n \xrightarrow{d} \tilde{Q}^-$, thus $\tilde{Q}_n \xrightarrow{d} \tilde{Q}$. □

2 Appendix B

We first illustrate the procedure involved in estimating the functions g_0 and h_0. The function $g(\mathbf{Z}) + h(\mathbf{Z})I_{\{E>\zeta\}}$ can be reorganized as $(1, I_{\{E>\zeta\}})(g(\mathbf{Z}), h(\mathbf{Z}))^\top$, and the log-likelihood function can be written as

$$\begin{aligned}
\ell_n(\boldsymbol{\eta}) :=& \frac{1}{n}\sum_{i=1}^n \Delta_i \log\left\{1-\exp\left\{-\Lambda(U_i)\exp\left\{\boldsymbol{\theta}^\top\tilde{\mathbf{X}}_i+\mathcal{I}_i(\zeta)^\top\mathcal{U}(\mathbf{Z}_i)\right\}\right\}\right\} \\
&-(1-\Delta_i)\Lambda(U_i)\exp\left\{\boldsymbol{\theta}^\top\tilde{\mathbf{X}}_i+\mathcal{I}_i(\zeta)^\top\mathcal{U}(\mathbf{Z}_i)\right\},
\end{aligned} \tag{16}$$

where $\mathcal{I}_i(\zeta) = \left(1, I_{\{E_i>\zeta\}}\right)^\top$, $\mathcal{U}(\mathbf{Z}_i) = (g(\mathbf{Z}_i), h(\mathbf{Z}_i))^\top$, $\boldsymbol{\theta} = (\boldsymbol{\beta}^\top, \boldsymbol{\gamma}^\top)^\top$ and $\tilde{\mathbf{X}}_i = \left(\mathbf{X}_i^\top, \mathbf{X}_i^\top I_{\{E_i>\zeta\}}\right)^\top$. As an illustration, a DNN structure with the width $\boldsymbol{p} = (4, 6, 6, 6, 2)^\top$ is shown in Figure 1. Obviously, in our case, the outputs are $g(\mathbf{Z})$ and $h(\mathbf{Z})$ respectively. For convenience in computation, the number of layers is set as 3, and the width for training $g(\mathbf{Z})$ and $h(\mathbf{Z})$ is set as 64, while the width in training the standard errors of $\boldsymbol{\beta}$ and $\boldsymbol{\gamma}$ is set as 50. The hyperparameters used to train in the simulation are obtained by a grid search, and the resulting hyperparameters maximize the full likelihood function on the test data, which are summarized as Table 1.

References

Bickle, P., Klaassen, C. A. J., Ritov, Y., and Wellner, J. A. (1993). *Efficient and Adaptive Estimation for Semiparametric Models.* Johns Hopkins Univ. Press.

Kosorok, M. R. (2008). *Introduction to empirical processes and semiparametric inference.* Springer Science and Business Media.

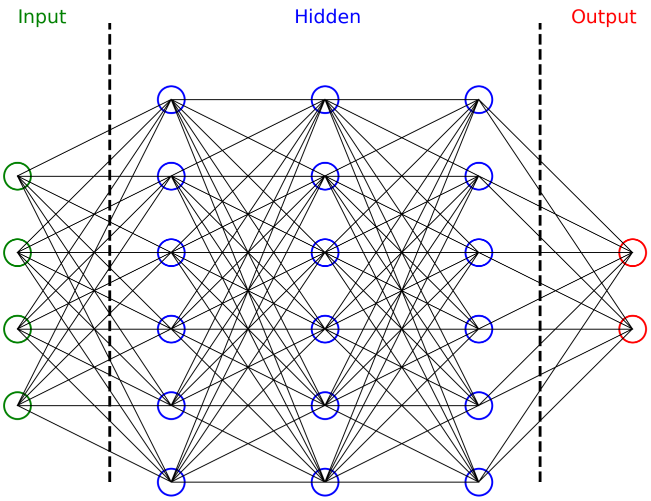

Figure 1: Visualization of a deep neural network with 3 hidden layers and the width $\boldsymbol{p} = (4, 6, 6, 6, 2)^\top$.

Table 1: Hyperparameters

		Model		SE ($\boldsymbol{\beta}$)		SE ($\boldsymbol{\gamma}$)	
	n	lr ($\times 10^{-4}$)	epoch	lr ($\times 10^{-4}$)	epoch	lr ($\times 10^{-4}$)	epoch
Case 1 (Linear)	1000	2.55	150	20	200	10	150
	2000	4.2	150	20	200	10	200
	4000	4.3	150	0.5	70	1	100
Case2 (Additive)	1000	3.7	180	8	150	7	150
	2000	5	160	10	150	15	150
	4000	6	225	0.7	100	3	100
Case 3 (Deep)	1000	3.2	200	10	200	10	200
	2000	4	200	10	150	10	200
	4000	5.5	200	0.8	50	2	100

Lu, M. G. and Zhang, Y. (2007). Estimation of the mean function with panel count data using monotone polynomial splines. *Biometrika*, 94(3):705–718.

Lu, X. W. and Song, P. X.-K. (2015). Efficient estimation of the partly linear additive hazards model with current status data. *Scandinavian Journal of Statistics*, 42(1):306–328.

Pons, O. (2003). Estimation in a cox regression model with a change-point according to a threshold in a covariate. *The Annals of Statistics*, 31(2):442–463.

Schmidt-Hieber, J. (2020). Nonparametric regression using deep neural networks with relu activation function. *The Annals of Statistics*, 48(4):1875–1897.

van der Vaart, A. W. (2000). *Asymptotic Statistics. Cambridge Series in Statistical and Probabilistic Mathematics 3.* Cambridge Univ. Press, Cambridge.

van der Vaart, A. W. and Wellner, J. A. (1996). *Weak Convergence and Empirical Processes: With Applications to Statistics. Springer Series in Statistics.* Springer, New York.

Zhong, Q. X., Müeller, J., and Wang, J. L. (2022). Deep learning for the partially linear Cox model. *The Annals of Statistics*, 50(3):1348–1375.
